# Insights into the molecular properties underlying antibacterial activity of prenylated (iso)flavonoids against MRSA

**DOI:** 10.1038/s41598-021-92964-9

**Published:** 2021-07-09

**Authors:** Sylvia Kalli, Carla Araya-Cloutier, Jos Hageman, Jean-Paul Vincken

**Affiliations:** 1 Laboratory of Food Chemistry, Wageningen University & Research, Wageningen, The Netherlands; 2 Biometris, Applied Statistics, Wageningen University & Research, Wageningen, The Netherlands

**Keywords:** Computational biology and bioinformatics, Drug discovery, Microbiology

## Abstract

High resistance towards traditional antibiotics has urged the development of new, natural therapeutics against methicillin-resistant *Staphylococcus aureus* (MRSA). Prenylated (iso)flavonoids, present mainly in the Fabaceae, can serve as promising candidates. Herein, the anti-MRSA properties of 23 prenylated (iso)flavonoids were assessed in-vitro. The di-prenylated (iso)flavonoids, glabrol (flavanone) and 6,8-diprenyl genistein (isoflavone), together with the mono-prenylated, 4′-*O*-methyl glabridin (isoflavan), were the most active anti-MRSA compounds (Minimum Inhibitory Concentrations (MIC) ≤ 10 µg/mL, 30 µM). The in-house activity data was complemented with literature data to yield an extended, curated dataset of 67 molecules for the development of robust in-silico prediction models. A QSAR model having a good fit (R^2^_adj_ 0.61), low average prediction errors and a good predictive power (Q^2^) for the training (4% and Q^2^_LOO_ 0.57, respectively) and the test set (5% and Q^2^_test_ 0.75, respectively) was obtained. Furthermore, the model predicted well the activity of an external validation set (on average 5% prediction errors), as well as the level of activity (low, moderate, high) of prenylated (iso)flavonoids against other Gram-positive bacteria. For the first time, the importance of formal charge, besides hydrophobic volume and hydrogen-bonding, in the anti-MRSA activity was highlighted, thereby suggesting potentially different modes of action of the different prenylated (iso)flavonoids.

## Introduction

*Staphylococcus aureus* (SA) and its oxacillin-resistant form (methicillin-resistant, MRSA) is one of the leading causes of healthcare-associated infections worldwide^1^. Furthermore, MRSA has also emerged as a major cause of community-associated and livestock infections^2^. In the United States, even though hospital-associated MRSA infections decreased by 5.4% from 2013 to 2016, community-associated infections increased by 1.6%^3^. In Europe, MRSA is a public health concern for Southern and Eastern European countries, in particular^4^. The World Health Organization has referred to MRSA as a high priority pathogen for the development of new therapeutics^5^. Over the last 30 years, only a few antibiotics have been approved as anti-MRSA agents, but MRSA has already developed resistance towards them^6^.

Novel chemical scaffolds with different modes of action to currently used antibiotics are constantly being investigated^7^. Prenylated flavonoids and isoflavonoids, collectively termed as (iso)flavonoids, as well as stilbenoids, have shown promising antibacterial activity against clinically-relevant pathogens, including MRSA^8,9^. Prenylated phenolic compounds are a class of secondary defence metabolites produced by species of the Fabaceae family upon (a)biotic stress^10^. Attachment of the prenyl moiety (3,3-dimethylallyl) to a phenolic skeleton is known to increase its antibacterial potency, due to the increased hydrophobicity conferred to the molecule^11^.

Several efforts have been made in correlating essential structural features of prenylated (iso)flavonoids with their anti-MRSA activity. Clear structure–activity relationships (SARs) remain difficult to be established due to the extensive structural diversity of prenylated (iso)flavonoids. This chemical diversity stems mainly from the different (iso)flavonoid subclasses (21 known to date). (Iso)flavones, (iso)flavanones, isoflavans, pterocarpans, pterocarpenes, 3-arylcoumarins, and 2-arylbenzofurans are some of the main (iso)flavonoid subclasses encountered in Fabaceae^10,12^. Chemical diversity increases further by the presence of substituents other than the prenyl group (hydroxyl, methoxyl), as well as the different configuration that the prenyl group can have (chain, pyran or furan). Therefore, SARs that apply to one subclass of prenylated (iso)flavonoids might not apply to another.

Methylation is one of the most common decorations of prenylated (iso)flavonoids. Methylation at the *C*7, for example, slightly reduces the anti-MRSA activity of prenylated isoflavones^13,14^, has no effect on the activity of prenylated isoflavans, while it was shown detrimental for prenylated coumarins^13^. Chain prenylation seems more favourable than ring prenylation in prenylated isoflavones with respect to anti-MRSA activity^14–16^. In contrast, the ring-prenylated pterocarpan, phaseollin (MIC 78 µM) is active, whereas its chain-prenylated analogue, phaseollidin has lower anti-MRSA activity (MIC 154 µM)^16^. Prenylation at *C*6 of isoflavones^14^ is better for anti-MRSA activity than *C*8-prenylation^13^, whereas the opposite seems to be preferred for flavanones^17^, consistent with the results for other G^+^ bacteria^18^.

Since prenylation-dependent hydrophobicity is not the only determinant of antibacterial activity, more systematic SARs are necessary to rationally portray how the overall molecular characteristics influence anti-MRSA activity. In silico tools, such as QSAR and pharmacophore modelling^19^ can aid the elucidation of these molecular characteristics. Properties, such as shape (flexibility and globularity) and surface (hydrophilic/hydrophobic regions) of prenylated (iso)flavonoids, have been shown to contribute to their antibacterial activity against the Gram-positive, *Listeria monocytogenes*^18^. The limitation of this QSAR analysis was the small dataset (30 molecules from 6 subclasses) used^18^, which did not allow splitting, thereby compromising the robustness of the models^20^. A (Q)SAR study on a larger dataset (67 (iso)flavonoids tested against SA or MRSA), which allowed splitting, has been performed by Sadgrove et al*.* (2020)^21^. However, this study considered molecules only from *Erythrina* species (Fabacae) including non-prenylated, prenylated, geranylated (iso)flavonoids and a chromen-4-one derivative. By using regression QSAR modelling, the importance of hydrogen-bonding, hydrophobicity, primary oxygens and charge distribution was highlighted in this study^21^. Nevertheless, the importance of these molecular properties with respect to the mechanism of action of these compounds was not addressed.

In this study, a multiple linear regression model was developed from an extensive, curated dataset of 67 prenylated (iso)flavonoids from 9 subclasses tested only against MRSA by using a QSAR approach. The model was externally validated with an additional set of 10 prenylated (iso)flavonoids and further assessed for its capacity to predict the level of activity of prenylated (iso)flavonoids against other Gram-positive bacteria (including SA, *Staphylococcus epidermis, Bacillus subtilis, Enterococcus facealis, and Streptococcus mutans*). In addition, a 3D pharmacophore model was developed to visualize the structural requirements for anti-MRSA activity. Both in-silico approaches were based on molecules from both in-house and literature activity data. For the in-house data, the activity (MIC values) of 23 prenylated compounds belonging to 6 subclasses was determined.

## Materials and methods

### Materials

Prenylated isoflavonoids (glabrene, 3′-hydroxy-4′-*O*-methyl-glabridin, 4′-*O*-methyl-glabridin, hispaglabridin A, hispaglabridin B, glyceofuran, glyceollidin II, glyceollin I, glyceollin II, glyceollin III, glyceollin IV, glyceollin V, dehydroglyceollidin II, dehydroglyceollin I, dehydroglyceollin II, dehydroglyceollin, III, dehydroglyceollin IV) and one prenylated flavone (glabrol) were previously purified and chemically characterized^22,23^. Wighteone, lupiwighteone, luteone, 2,3-dehydrokievitone, licoisoflavone A, neobavaisoflavone, iso-osajin and 6,8-diprenygenistein were purchased from Plantech UK (Reading, UK). Isowighteone and anhydrotuberosin were purchased from ChemFaces (Wuhan, Hubei, China). 6-Prenylnaringenin and ampicillin were purchased from Sigma Aldrich (St. Louis, MO, USA). Psoralidin and 8-prenylnaringenin were purchased from Sanbio B.V. (Uden, The Netherlands). Glabridin was purchased from Wako (Osaka, Japan). Bacto brain heart infusion (BHI) broth was purchased from BD (Franklin Lakes, NJ, USA), tryptone soya broth (TSB) and bacteriological agar from Oxoid Ltd (Basingstoke, UK), and peptone physiological salt solution (PPS) from Tritium Microbiologie (Eindhoven, The Netherlands). Ethanol absolute (EtOH) was purchased from Biosolve (Valkenswaard, The Netherlands).

### Methods

#### Antibacterial susceptibility assay

Different prenylated (iso)flavonoids were tested for their antibacterial activity against MRSA 18HN (strain kindly provided by RIVM, Bilthoven, The Netherlands). Bacteria were streaked from a − 80 °C glycerol stock onto a BHI agar plate and incubated 24 h at 37 °C. Next, one colony was transferred to BHI broth (10 mL) and further incubated for 18 h at 37 °C. These overnight cultures were diluted with TSB (final inoculum concentration 3.8 ± 0.4 log CFU/mL). Stock solutions of the different prenylated compounds were prepared in aqueous EtOH (70% v/v) or DMSO and subsequently diluted with TSB. A series of concentrations were tested ranging from 3.1 to 100 or 150 µg/mL (2.1% v/v solvent max.) of prenylated (iso)flavonoids. No solubility issues were associated with prenylated (iso)flavonoids in hydrophilic growth media (TSB) at any of the concentrations tested. Equal volumes (100 µL) of bacteria and prenylated compound solutions in TSB were mixed into a 96-well plate. The 96-well plate was incubated in a SpectraMax M2e (Molecular Devices, Sunnyvale, CA, USA) at 37 °C with constant linear shaking. The optical density (OD) at 600 nm was measured every 5 min for 24 h.

A positive control (vancomycin, 2 µg/mL), negative controls (TSB suspension of bacteria with and without 2.5% (v/v) max. solvent) and blanks (compounds and TSB medium without bacteria) were used for optical comparison and sterility control. Growth inhibition was assessed by measuring the time to detection (TTD), i.e. the time to reach a change in OD of 0.05 units^24^. When no change in OD (i.e. ΔOD < 0.05) was observed after the 24 h of incubation, cell viability was confirmed by plate counting, as described by Araya-Cloutier et al. (2018)^18^. The minimum inhibitory concentration (MIC) and the minimum bactericidal concentration (MBC) were defined as described elsewhere^18^. Prenylated compounds were tested in two independent biological reproductions, each performed in duplicate.

#### Inactivation kinetics

An overnight MRSA culture was diluted to 4.4 ± 0.5 Log (CFU/mL) and mixed with prenylated phenolics at their MIC and 2xMIC. Samples were incubated in duplicate at 37 °C/125 rpm. At different time points (0, 2, 4, 6, 24 h), 100 µL of culture medium were taken and decimally diluted in PPS. Dilutions were spread on BHI agar plates and incubated overnight at 37 °C, after which colonies were counted. Vancomycin and ampicillin at 2 µg/mL were used as control antibiotics.

#### QSAR modeling

##### Dataset construction

An outline of the different steps followed for the QSAR modelling is demonstrated in Fig. S1. First, 106 prenylated (iso)flavonoids together with their anti-MRSA activity were mined from 19 different studies (2000–2017, Table S1). These studies where chosen under the premise that agar or broth (micro)dilution assays were used to determine the anti-MRSA activity. The activity data of prenylated (iso)flavonoids from 16 studies was combined with the activity data of the prenylated (iso)flavonoids generated in-house, to obtain a dataset of 94 molecules with reported MIC values. The activity data from the 3 other, randomly chosen, studies^25–27^ was used to externally validate the model^28^. The choice for the construction of this independent external validation set was based on whole studies, instead of various molecules from different studies to minimize extreme experimental variability. Compounds for which no specific MIC value was obtained (i.e. MIC was higher than the highest tested concentration), were excluded from the QSAR modelling study. When more than one MRSA strain was tested and a range of MIC values was given, or more than one MIC value per compound was available, the highest MIC value was used to consider the worst-case scenario. Activity data [MIC values (µM)] was converted to pMIC [− log MIC (M)] (to improve the normality of the data distribution).

##### Dataset curation

A defined dataset of 76 prenylated (iso)flavonoids with established MICs was intended to enter the training/test phase. To ensure uniform chemical diversity upon the splitting of the modelling set, two selection criteria were applied for the compounds used for the QSAR modelling. First, (iso)flavonoids with common chain prenylation (i.e. 3,3-dimethylallyl) and ring prenylation (2″-isopropenylfuran and/or 2,2-dimethylpyran) were exclusively considered (**criterion 1**). Second, for each subgroup of mono- and di-prenylated compounds (in each subclass) two representative compounds had to be present, otherwise the subgroup was excluded (**criterion 2**). Chemical structures were extracted from literature and inputted to the modelling software (Molecular Operating Environment, MOE, v.2019.08, Chemical Computing Group, Montreal, QC, Canada) using the canonical SMILES codes from PubChem. If not available, the chemical structure was drawn manually using the PubChem sketcher to obtain the SMILES code before importing to MOE. Structures were energy minimized by using the molecular orbital package (MOPAC) PM3 forcefield at a root mean square (RMS) gradient of 0.01 kcal/mol/Å^2^. Then, a conformational search was performed by using LowModeMD (RSM gradient 0.1 kcal/mol/Å^2^, other settings were left on default) was performed for all compounds in the database.

Optimized chemical structures were used to calculate different molecular descriptors available in MOE. After eliminating descriptors that were identical for all molecules or redundant descriptors (inter-correlation R_pearson_ > 0.99), a total number of 120 descriptors were finally incorporated into the database (list of descriptors in Table S2). Statistical modelling was performed using R (R Core Team. 2021^29^) and a combination of in-house created scripts and specific libraries mentioned where applicable. After the two selection criteria were applied, the modelling set of 70 complying compounds was split into a training set (80%, for model development) and test set (20%, for model selection based on model’s predictive power on the test set)^28^ using the Kennard–Stone algorithm from the R-package ‘prospectr’ (using principal components and retaining 95% explained variance)^30^. This procedure selects the best chemically representative subset as a training set, avoiding overoptimistic splitting which might occur during random splitting^31^.

##### Model development and validation

A genetic algorithm (GA) (R-package ‘GA’, version 3.2) was used to select a small subset of descriptors that best predict the antibacterial activity (pMIC). pMIC activity was modelled using multiple linear regression (MLR) using the selected descriptors. Model accuracy for a given set of predictors was determined by a leave-one-out cross validation (LOOCV) procedure and calculated as Q^2^_LOO_. Every GA run was repeated 12 times with different starting seeds. Combinations of predictors with a variance inflation factor (VIF) > 5 (calculated with R-package ‘car’), indicating a strong inter-correlation, were effectively removed from the GA population by penalizing the fit during the GA run. GA parameters were optimised using a full factorial experimental design and were found to be population size = 150, cross-over rate = 0.6, mutation rate = 0.3. The maximum number of iterations was set to 200 and elitism set to 8. The number of predictors to be selected during a GA run was fixed and varied between 2 and 7.

The applicability domain (AD) of the models was calculated by means of the William’s plot. If the first GA run indicates the presence of outliers with high leverage then all outliers (including high residual outliers) are removed from the dataset and the GA is repeated until no high leverage outliers are detected. High leverage molecules are poorly fitting, highly impactful molecules that force the GA to accommodate them in the model, having great impact on its performance^32^. In our study, after the first GA-run, two molecules, glyceollin I (**87**) and glyceollin III (**89**) (Table S1) were flagged as high leverage molecules. Erysubin F (**55**) was highlighted as a high residual outlier. Erysubin F is reported to have an outstandingly low activity (MIC 100 µg/mL) for a double prenylated isoflavone^33^. All three molecules were removed from the dataset, the GA-MLR procedure was repeated once more and a new AD (AD’) was defined.

The final dataset used for the development and selection of the best QSAR model comprised 67 prenylated (iso)flavonoids from 9 different (iso)flavonoid subclasses: 2-arylbenzofurans, 3-arylcoumarins, flavanones, isoflavans, isoflavanones, isoflavenes, isoflavones, pterocarpans and pterocarpenes.

Several internal and external statistical parameters which are typically used to assess the statistical performance of the models were calculated. Some of the internal ones include: the significance of the models and the descriptors individually (*p* value < 0.05), the coefficient of determination, R^2^, and the adjusted R^2^ (R^2^_adj_) which corrects for the difference in the number of descriptors, and the leave-one-out cross-validation coefficient of determination (Q^2^_LOO_) which is used to assess the model’s internal predictivity. According to Tropsha et al*.* (2010), the R^2^ and Q^2^_LOO_ should be > 0.6 and > 0.5, respectively^34^. Furthermore, the maximum variance inflation factor (VIF_max_), which indicates potential inter-correlation of the descriptors in the generated models, should preferably be < 5^35^. The accuracy of the prediction of the test set was assessed by calculating the Q^2^_F3_ for the test set (Q^2^_test_) according to Consonni et al*.* (2009), a calculation based on the number of training objects^36^. For both sets, the % of prediction error was calculated with the following formula: (pMIC_observed_ − pMIC_predicted_)/pMIC_observed_ * 100. Last, the best QSAR model was further evaluated with the external validation set; an independent set of 10 compounds with established MICs verified to belong to the applicability domain of the selected model (AD′). The aim of this external validation set was to simulate the scenario where new prenylated (iso)flavonoids with unknown activity are predicted by the model. Based on the small number of datapoints and the narrow range of activities, model’s prediction accuracy on the external validation set was assessed by determining the prediction error percentage and the mean absolute errors (MAE). An acceptable prediction error percentage was considered when this was less than 10% which corresponds to maximum one twofold dilution difference between predicted and experimental values^37,38^. MAE was calculated using the following formula: 1/n * ∑|pMIC_observed_ − pMIC_predicted_| with n being the number of external validation objects. If MAE is up to 10% of the training set range, then it is considered as a good prediction whereas if it is more than 15% it is considered a bad one, according to *Roy *et al*.* (2016)^39^.

##### Prediction of activity of prenylated (iso)flavonoids against other Gram-positive bacteria

The best QSAR model for MRSA was also used to predict the level of activity (low, moderate, high) of prenylated (iso)flavonoids tested against other Gram-positive bacteria. Thus, the antibacterial activity of 71 prenylated (iso)flavonoids tested against other Gram-positive bacteria was mined from literature (studies from 1988 to 2020). The compounds were gathered under the premise that they fall within the applicability domain of our QSAR-MRSA model. Furthermore, only compounds from the same subclasses as the ones used to construct the QSAR-MRSA were used, to avoid extreme extrapolation of the model. When the compounds were tested against different Gram-positive bacteria or when more than one MIC was available per compound, then the highest MIC was taken into account to consider the worst-case scenario.

The quality of prediction was assessed by verifying that the compounds were still classified as active (MIC ≤ 25 μg/mL), moderately active (25 < MIC < 100 μg/mL) or inactive (MIC ≥ 100 μg/mL) after the prediction. Based on the nature of the MIC determination method (agar or broth dilution assays), misclassified compounds for which their predicted activity differed from their experimentally measured activity by one two-fold dilution (acceptable experimental error in dilution assays^37,38^), were considered as correctly predicted.

#### Pharmacophore elucidation

A pharmacophore model was built using the pharmacophore elucidation query module of MOE. Since no definite target site of these compounds is known, a ligand-based pharmacophore methodology was employed. This is performed by aligning the different active ligands and determining the essential common chemical queries necessary for activity. All 77 compounds (curated training, curated test and external validation set) were used to extract the pharmacophore features. An activity threshold of pMIC ≥ 4.1 (MIC ≤ 25 μg/mL) was set to distinguish the active from the moderate/inactive antibacterials. The quality of the model was assessed based on its capacity to discriminate the active from the moderately active or inactive molecules. The overall accuracy refers to the percentage of correct predictions, including both actives and inactive molecules. The positive accuracy refers to the proportion of correctly predicted actives, indicating the sensitivity of the model. The negative accuracy is calculated based on the percentage of correctly predicted inactives showing the specificity of the model.

## Results

### Experimental anti-MRSA activity of prenylated (iso)flavonoids

Table 1 shows the antibacterial potency of prenylated (iso)flavonoids against MRSA 18HN. The classification of the compounds with respect to their anti-MRSA activity was based on literature of antimicrobial phytochemicals^40^. Compounds with MIC values ≤ 25 µg/mL were considered active, with most active being the ones having a MIC of ≤ 10 µg/mL. Compounds with MIC values between 25 and 100 µg/mL were considered moderately active, whereas those with MIC values ≥ 100 µg/mL were classified as inactive. The most active compounds experimentally tested in this study were the isoflavone, 6,8-diprenylgenistein (**50**) and the di-prenylated flavanone, glabrol (**13**) which showed a MIC of 9 µg/mL, corresponding to 23 µM and 24 µM, respectively. These compounds share double chain prenylation in their backbones (Fig. S2). The most active mono-prenylated compound was the ring-prenylated isoflavan, 4′-*O*-methylglabridin (**17**) with a MIC of 10 µg/mL (30 µM). Inactive prenylated (iso)flavonoids with an established MIC tested were glyceollins I–III (**87**–**89**) (> 100 µg/mL, > 296 µM). The mono-prenylated compounds (**51, 63, 68, 76, 85, 91, 101**) and the di-prenylated hispaglabridin B (**26**) had negligible antibacterial activity against MRSA (TTD similar to that of the control at 100 µg/mL, therefore MIC ≫ 100 µg/mL) (Table S1).

**Table 1:** Minimum inhibitory concentration (MIC) and minimum bactericidal concentration (MBC) of prenylated (iso)flavonoids tested in this study against MRSA 18HN.

Subclass	Name	MIC μg/mL [μM]	MBC μg/mL [μM]
Flavanones	6-Prenylnaringenin (**12**)	38 [110]	44 [129]
	Glabrol (**13**)	9 [24]	19 [48]
	Sophoraflavanone B (**14**)	22 [64]	25 [73]
Isoflavans	3′-OH-4′-*O*-Methylglabridin (**16**)	16 [44]	26 [73]
	4′-*O*-Methylglabridin (**17**)	10 [30]	23 [66]
	Glabridin (**22**)	13 [39]	19 [58]
	Hispaglabridin A (**25**)	44 [111]	44 [111]
Isoflavones	6,8-Diprenylgenistein (**50**)	9 [23]	16 [38]
	Isowighteone (**64**)	22 [65]	34 [102]
	Licoisoflavone A (**65**)	25 [71]	50 [141]
	Luteone (**69**)	25 [71]	44 [123]
	Neobavaisoflavone (**70**)	38 [116]	50 [155]
	Wighteone (**74**)	16 [46]	22 [65]
Isoflavene	Glabrene (**49**)	25 [78]	44 [136]
Pterocarpans	Glyceollidin II (**86**)	44 [129]	44 [129]
	Glyceollin I (**87**)	100 [296]	n.a
	Glyceollin II (**88**)	150 [443]	n.a
	Glyceollin III (**89**)	100 [296]	n.a
	Glyceollin IV (**90**)	44 [123]	75 [212]
Pterocarpenes	Dehydroglyceollidin II (**98**)	22 [68]	22 [68]
	Dehydroglyceollin I (**99**)	16 [49]	22 [68]
	Dehydroglyceollin II (**100**)	19 [59]	19 [59]
	Dehydroglyceollin IV (**102**)	44 [130]	50 [149]

**Figure 1:**
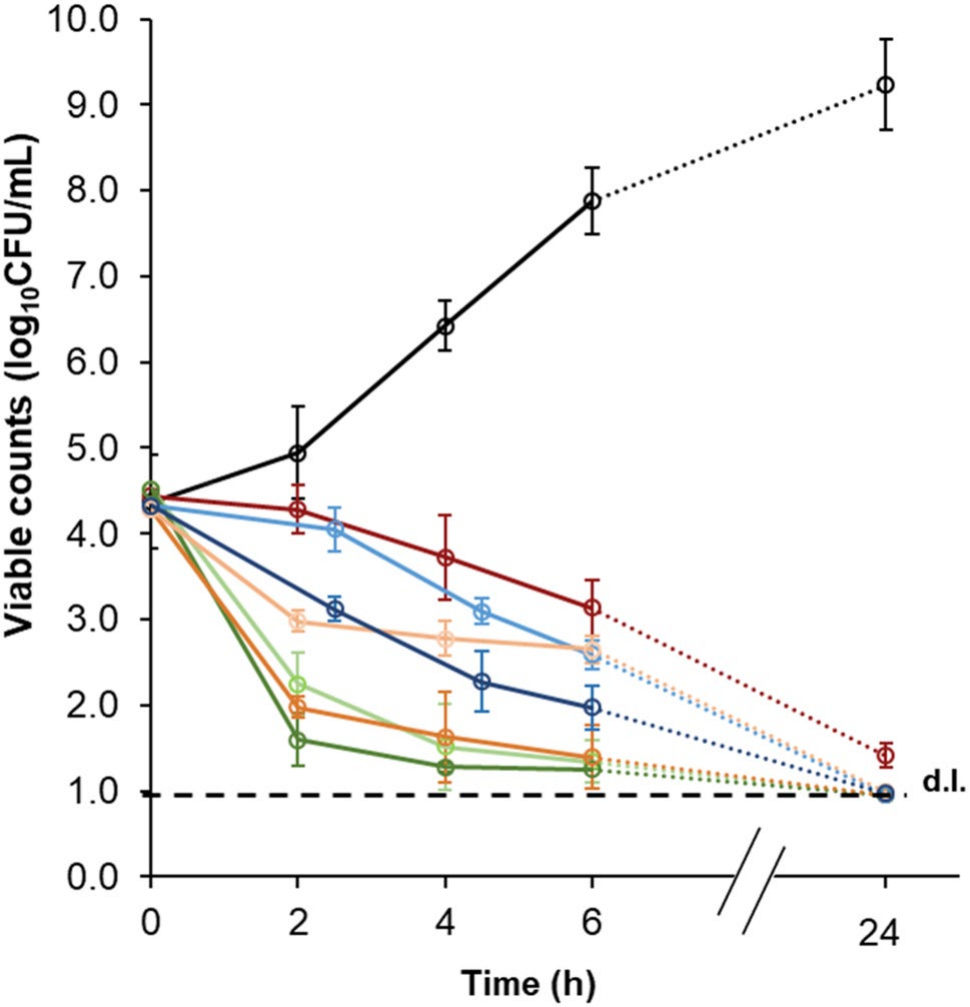
MRSA 18HN inactivation kinetics in the presence of the three most antibacterial prenylated (iso)flavonoids tested in this study, at their MIC (light shade) and 2xMIC (dark shade). Control (black), vancomycin (2 µg/mL, red), 4′-*O*-methylglabridin (**17**) (blue), glabrol (**13**) (orange) and 6,8-diprenylgenistein (**50**) (green).

Figure 1 shows the inactivation kinetics of MRSA 18HN in the presence of the top three antibacterial prenylated (iso)flavonoids tested in this study. The di-prenylated (iso)flavonoids, 6,8-diprenylgenistein (**50**) and glabrol (**13**) inactivated MRSA by > 99% in the first 2 h of contact at 2 × their MIC, whereas the mono-prenylated isoflavan, 4′-*O*-methyl-glabridin (**17**) inactivated MRSA after 6 h also at 2 × its MIC. The control antibiotic vancomycin at its MIC decreased the initial inoculum by > 99% only after 24 h. The slow action of vancomycin against MRSA is in line with what has been shown before for MRSA and *Streptococcus pneumoniae*^41, 42^.

### Extended prenylated (iso)flavonoid dataset for QSAR modelling of anti-MRSA activity

A QSAR study was performed to pinpoint overall molecular properties contributing to the anti-MRSA activity. To construct robust and reliable QSAR models, the experimental data shown above was combined with literature data to enlarge and diversify our collection of prenylated (iso)flavonoids. In total, 76 prenylated (iso)flavonoids with established MICs from 10 subclasses were collected (Table S1). This diverse set of compounds was curated (see criteria 1 and 2 in **Dataset construction**), which resulted in 70 molecules in the modelling set (Fig. S1). During the training phase, genetic algorithm (GA) flagged glyceollin I (**87**), glyceollin III (**89**) and erysubin F (**55**) as outliers. Thus, QSAR modelling was performed based on a curated modelling set 67 of molecules from 9 subclasses. The best model was further externally validated with a set of 10 molecules from three studies independent from those used in the modelling set^28^.

Similar to what was observed for our in-house data, more than 90% of the di-prenylated (iso)flavonoids compiled from literature, showed a high anti-MRSA activity (MIC ≤ 25 µg/mL, 8–64 μM), whereas half of the mono-prenylated ones were active. Structures of representative active anti-MRSA agents per isoflavonoid subclass derived from the extended dataset can be found in Fig. S3.

The most powerful anti-MRSA di-prenylated (iso)flavonoids were isolupalbigenin, erybraedin A and eryvarin W (**62**, **78**, and **106**, Table S1) with MIC = 3 µg/mL, 8 μM. These molecules have one prenyl-group on the A-ring and one on the B-ring (Fig. S3). The most active mono-prenylated isoflavonoid was orientanol B (**93**) with MIC = 6 µg/mL, 18 μM (Table S1). This molecule showed comparable activity to the in-house tested mono-prenylated isoflavan, 4′-*O*-methyl-glabridin (**17**) with MIC = 10 µg/mL, 30 μM. Both compounds are prenylated on the A-ring and possess the same type and amount of substituents (one hydroxy-group and one methoxy-group), yet in different positions over the backbone (Fig. S3).

### QSAR model development, selection and external validation

The best GA-MLR-models obtained per number of descriptors and their statistical performance are listed in Table 2.

**Table 2:** Best GA-MLR models for predicting the antibacterial activity of prenylated (iso)flavonoids against MRSA.

n	Equation	R^2^	R^2^_adj_	Q^2^_LOO_	Q^2^_test_
2	$y = 2.787\text{}\left( {0.331} \right) - 0.159\text{}\left( {0.048} \right)\text{*}h\_emd\_C + 0.006\text{}\left( {0.001} \right)\text{*}vsurf\_D4$	0.56	0.54	0.51	0.73
3	$y = 1.885\text{}\left( {0.305} \right) - 0.701\text{}\left( {0.183} \right)\text{*}h\_pavgQ + \text{}0.006\text{}\left( {0.001} \right)\text{*}vsurf\_D4 - 0.054\text{}\left( {0.018} \right)\text{*}vsurf\_IW7$	0.60	0.57	0.53	0.78
4	$y = 2.551\text{}\left( {0.343} \right)\,- \,0.178\text{}\left( {0.045} \right)\text{*}h\_emd\_C\, +\, 0.006\text{}\left( {0.001} \right)\text{*}vsurf\_D4\, -\, 0.050\text{}\left( {0.017} \right)\text{*}vsurf\_IW7\, +\, 0.047\text{}\left( {0.016} \right)\text{*}E\_vdw$	0.64	0.61	0.57	0.75
5	$y = 6.240\text{}\left( {0.270} \right) + 0.030\text{}\left( {0.006} \right)\text{*}PEOE\_VSA\_PPOS - 2.521\text{}\left( {0.275} \right)\text{*}vsurf\_CW3 - 0.061\text{}\left( {0.020} \right)\text{*}vsurf\_IW7 - 0.839\text{}\left( {0.238} \right)\text{*}h\_pavgQ + 0.011\text{}\left( {0.006} \right)\text{*}PEOE\_VSA+\emph{2}$	0.68	0.64	0.60	0.64
6	$y = 6.207\text{}\left( {0.262} \right) + 0.027\text{}\left( {0.006} \right)\text{*}PEOE\_VSA\_PPOS - 2.558\text{}\left( {0.267} \right)\text{*}vsurf\_CW3 - 0.063\text{}\left( {0.019} \right)\text{*}vsurf\_IW7 - 0.888\text{}\left( {0.214} \right)\text{*}h\_pavgQ + 0.011\text{}\left( {0.005} \right)\text{*}PEOE\_VSA+\emph{2} + 0.025\text{}\left( {0.012} \right)\text{*}vsurf\_DD12$	0.69	0.65	0.61	0.65
7	$\text{y} = 3.530\left( {1.470} \right) + 0.030\left( {0.006} \right)\text{*}\text{}PEOE\_VSA\_PPOS - 2.690 \left( {0.270} \right)\text{*}vsurf\_CW3 - 0.077 \left( {0.020} \right)\text{*}vsurf\_IW7 - \text{0.878}\text{(0.208)*}h\_pavgQ + \text{0.017}\text{(0.006)*}PEOE\_VSA+\emph{2} + 0\text{.030}\text{(0.012)*}vsurf\_DD12 - \text{0.317}\text{(0.171)*}PM3\_IP$	0.71	0.67	0.60	0.67

n: number of descriptors; R^2^: coefficient of determination (> 0.6)^31^; R^2^_adj_: adjusted R^2^; Q^2^_LOO_: leave-one-out cross-validated coefficient of determination (> 0.5)^31^; Q^2^_test_: correlation coefficient for the test set. All models were significant (*p* values < 1.0e^−7^) and no multicollinearity of descriptors was observed (VIF_max_ < 3.2)^32^. The F statistics for all models were > 17 with the largest *p* value being 2.1e^−10^. Values in parentheses refer to the standard error of the regression coefficients of the models.

**Figure 2:**
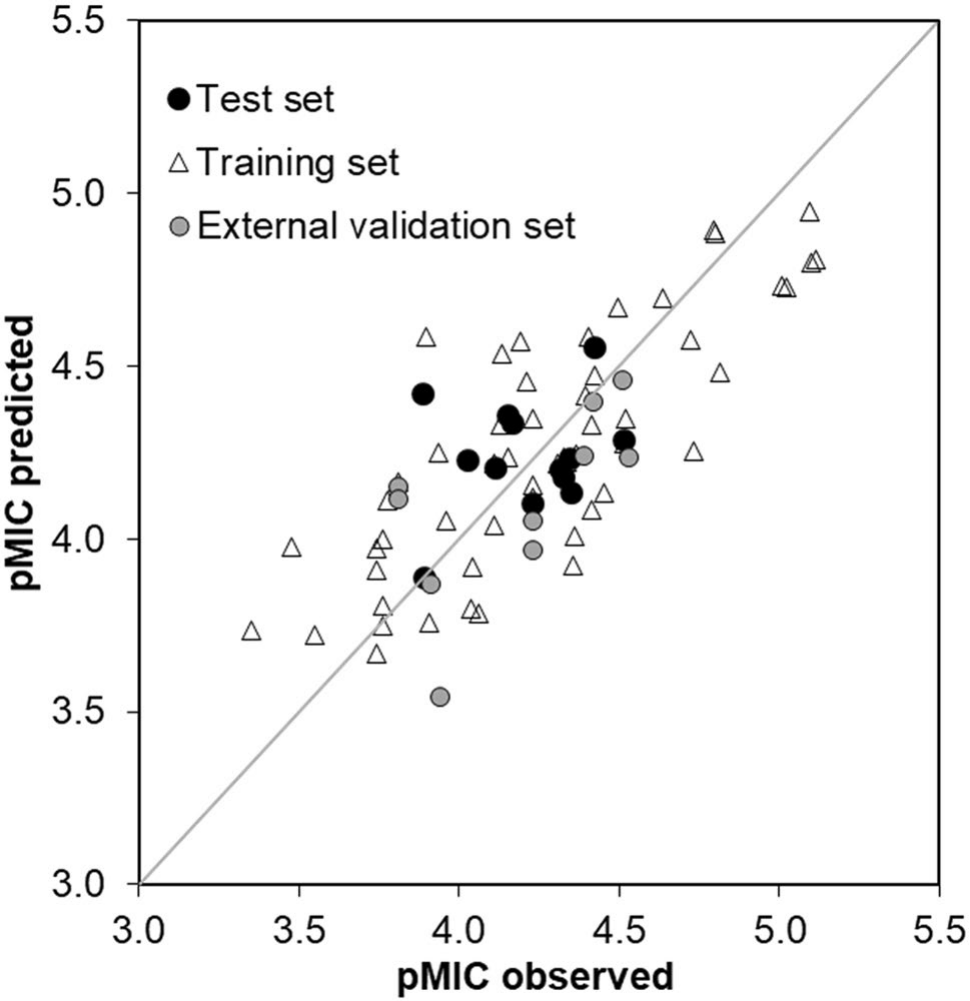
Correlation of the experimental and predicted anti-MRSA activity (pMIC, M) of prenylated (iso)flavonoids by using our best 4-descriptor QSAR model for the training, the test and the external validation sets. The applicability domain of the QSAR model can be found in Fig. S4.

Models with four or more descriptors complied with the thresholds of all validation parameters of the QSAR models, whereas models with less descriptors (2–3) gave relatively poor adjusted R^2^’s (R^2^_adj_). Usually, a higher number of descriptors results in over-fitted/more complex, less interpretable models. Thus, we chose the four-descriptor model as the best, since it balances well statistical validity, predictivity of the training (Q^2^_LOO_ 0.57 and average prediction error of 4%, Table S1) and the test set (Q^2^_test_ 0.75 and average prediction error of 5%, Table S1) (Fig. 2) and model’s interpretability. The model was also externally validated by predicting an independent set of 10 compounds (**1, 19, 28–30, 36, 37, 44, 46, 47**) coming from three studies^25–27^ and low average prediction errors (5%) were obtained (Table S1). The mean average error (MAE) of the external validation set was calculated to be 11.5% being marginally above the 10.0% threshold for good predictions reported by Roy et al*.* (2016)^39^. The applicability domain of the best 4-descriptor model can be found in Fig. S4.

In the chosen four-descriptor model, the hydrophobic volume at the 4th energetic level, i.e. − 0.8 kcal/mol (*vsurf_D4*) was the most significant descriptor (*p* value 2e^−10^), followed by the sum of hydrogen bond donor strengths of carbon atoms (*h_emd_C*, *p* value 2e^−4^). In addition, the unbalance between the centre of mass of a molecule and the position of the hydrophilic regions around it (*vsurf_IW7)* and the van der Waals interaction energy (*E_vdw)* were the least significantly correlated descriptors (*p *value 6e^−3^). Definitions of the descriptors can be found in Table S2.

### Predicting the level of antibacterial activity of prenylated (iso)flavonoids against other Gram-positive bacteria

The QSAR model developed for MRSA was additionally assessed for its capacity to predict the level of activity (low, moderate, high) of prenylated (iso)flavonoids tested against other Gram-positive bacteria (Table S3). Thus, activity data (MIC values) of 71 prenylated (iso)flavonoids tested against SA, *Staphylococcus epidermis, Bacillus subtilis, Enterococcus facealis* and *Streptococcus mutans* was collected (Table S3 and Fig. S5). The model predicted correctly the level of activity of 73% of the compounds (Fig. S6). This shows that the model is robust enough to be used as a tool for quick screening for potent antibacterial prenylated (iso)flavonoids. Half of the incorrectly predicted molecules were flavanones (Table S3 and Fig. S5), possibly due to the limited structural variation of flavanones in the training and test set (Table S1) used to develop the model.

### Interpretation of most frequently used descriptors in statistically compliant models

**Figure 3:**
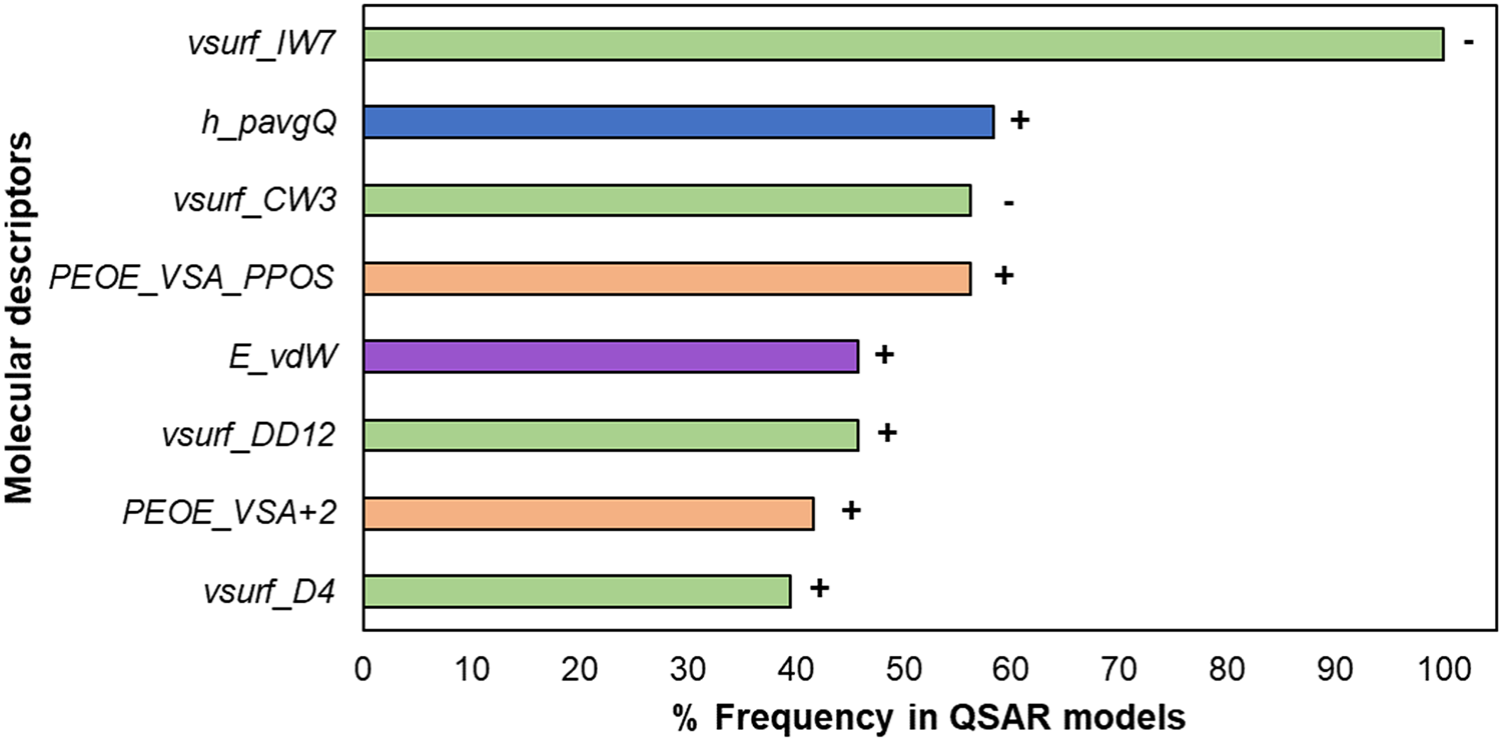
Descriptors most frequently selected by the GA to predict the anti-MRSA activity. The positive (+) or negative (−) correlation of each descriptor with anti-MRSA activity is shown next to the bars. Colour coding is used to indicate the different descriptor families. Green indicates the surface area, volume and shape (*vsurf*) descriptors, blue indicates the Hückel-theory (*h*) descriptor, orange represents the partial charge descriptors (*PEOE_VSA*) and purple is used to indicate the energy descriptor. Definitions of the descriptors can be found in Table S2.

To obtain a deeper insight into the molecular properties essential for activity, we analysed the most frequently used descriptors, i.e. present in more than 40% of the statistically compliant models (R^2^_adj_ > 0.6 and Q^2^_LOO_ > 0.5)^34^ (Fig. 3).

#### Hydrophobic volume and balanced hydrophilic fraction favour anti-MRSA activity

The most frequently used descriptor family included the surface area, volume and shape descriptors, *vsurf* (green colour in Fig. 3). These descriptors were calculated on multiple energetic levels, as volume and shape are influenced by the energy of the molecules^43^.

The descriptor *vsurf_IW7* was present in all statistically compliant models. It represents the hydrophilic integy moment at − 5.0 kcal/mol, an energy level known to be representative for polar and H-bond donor and acceptor regions^43^. It shows the distribution of hydrophilic moieties over the molecules by measuring the unbalance between the hydrophilic moieties and the centre of molecular mass. Its negative correlation to the anti-MRSA activity suggests that the hydrophilic regions close to the centre of mass or balanced at opposite directions in the molecule favour the activity^44^.

The descriptor *vsurf_CW3* quantifies the ratio between the hydrophilic surface and the total molecular surface^43^ and was one of the most strongly correlated descriptors (4.7e^–13^–4.1e^–09^, Table S4). Its negative contribution to the anti-MRSA activity indicates that a small hydrophilic fraction favours anti-MRSA activity. All molecules with *vsurf_CW3* of < 1.043 Å^3^ possessed high activity (except dehydroglyceollin IV (**102**) and hispaglabridin A (**25**)).

The *vsurf_DD12* descriptor quantifies the contact distance of lowest and the 2nd lowest hydrophobic energy points of a molecule. Its positive, although weak contribution to activity (*p* value, 4.7e^−02^–1.5e^−01^, Table S4) implies that the hydrophobically interacting groups or regions should be far away from each other and not localized closely together in the molecule^45^.

Last, the *vsurf_D4* descriptor is a measure of the hydrophobic volume at an energy level of − 0.8 kcal/mol (known to account for polarizability and dispersion forces)^43^. This descriptor was positively correlated to activity indicating that larger hydrophobic surfaces are preferred.

#### (Partial) charges favour anti-MRSA activity of prenylated (iso)flavonoids

##### Formal positive charge

The second most frequently used, positively correlated descriptor in the QSAR models was the Hückel theory descriptor, *h_pavgQ* (blue colour in Fig. 3). Hückel theory descriptors take into account local resonance and electron withdrawing effects^46^. This descriptor refers to the average total (formal) charge sum across protonation states at pH 7^47^, calculated based on the relative concentration of various protonation states of the molecule^48^. Isoflavones, (iso)flavanones and 3-aryl coumarins had generally higher values for this descriptor. Possibly, the presence of the carbonyl group on the C-ring of these subclasses enhances the acidity of hydroxyl protons (at *C*7 and *C*5) due to stabilization of the negative charge by resonance. In contrast, pterocarpans, pterocarpenes and isoflavans, lacking this carbonyl group, had low *h_pavgQ* values. Examples of the correlation of *h_pavgQ* with the activity can be found in Fig. S7.

##### Partial positive charge

The 2D partial charge descriptors, *PEOE_VSA_PPOS* and *PEOE_VSA* + *2* were frequently used, positively correlated variables in the QSAR models (orange in Fig. 3). *PEOE_VSA* descriptors are calculated based on the Partial Equalization of Orbital Electronegativities method and capture electrostatic interactions^49^. *PEOE_VSA_PPOS* (*p* value 3.6e^−06^ to 1.4e^−05^, Table S4) refers to the total positive polar van der Waals surface area (in Å^2^), where the partial charge is greater than + 0.200e. In fact, this descriptor depends on the number of oxygen atoms within the molecule^50^. Isoflavones, isoflavanones and 3-aryl coumarins were the most oxygenated subclasses in the dataset and showed large, partially positive surface areas within the molecule (*PEOE_VSA_PPOS* > 35 Å^2^). *PEOE_VSA* + *2* (*p *value 4.2e^−03^ to 1.6e^−01^, Table S4) refers to the sum of van der Waals (vdw) surface area for each atom (in Å^2^) of which the partial charge is between + 0.100e and (including) + 0.149e. Based on the level of significance of these two *PEOE* descriptors, small partial positive charges are less determinant of anti-MRSA activity compared to larger partial positive charges.

#### Van der Waals surface energy

The energy descriptor *E_vdw* (purple colour in Fig. 3) was also used as a positively correlated explanatory variable. This descriptor represents the van der Waals (vdW) component of the potential energy of the molecules^51^. Pterocarpans, pterocarpenes and 2-arylbenzofurans had clearly lower vdW interaction energies (*E_vdW* < 11.1 kcal/mol) than the rest of the subclasses. Pterocarpans and pterocarpenes are more rigid molecules than the other (iso)flavonoid subclasses due to the presence of an additional D-ring^18^. This higher rigidity should facilitate vdW interactions, due to lower entropic penalties on the rigid molecules compared to more flexible ones^52^. 2-Arylbenzofurans, despite the lack of the extra D-ring, contain a furan C-ring, which contributes to increased molecular planarity compared to a pyran^53^ (Fig. S8). Thus, the more rigid or planar a molecule is, the lower the vdW interaction energy and the lower the activity. This implies that other interactions, most likely electrostatic, are more important for activity.

### Pharmacophore modelling

**Figure 4:**
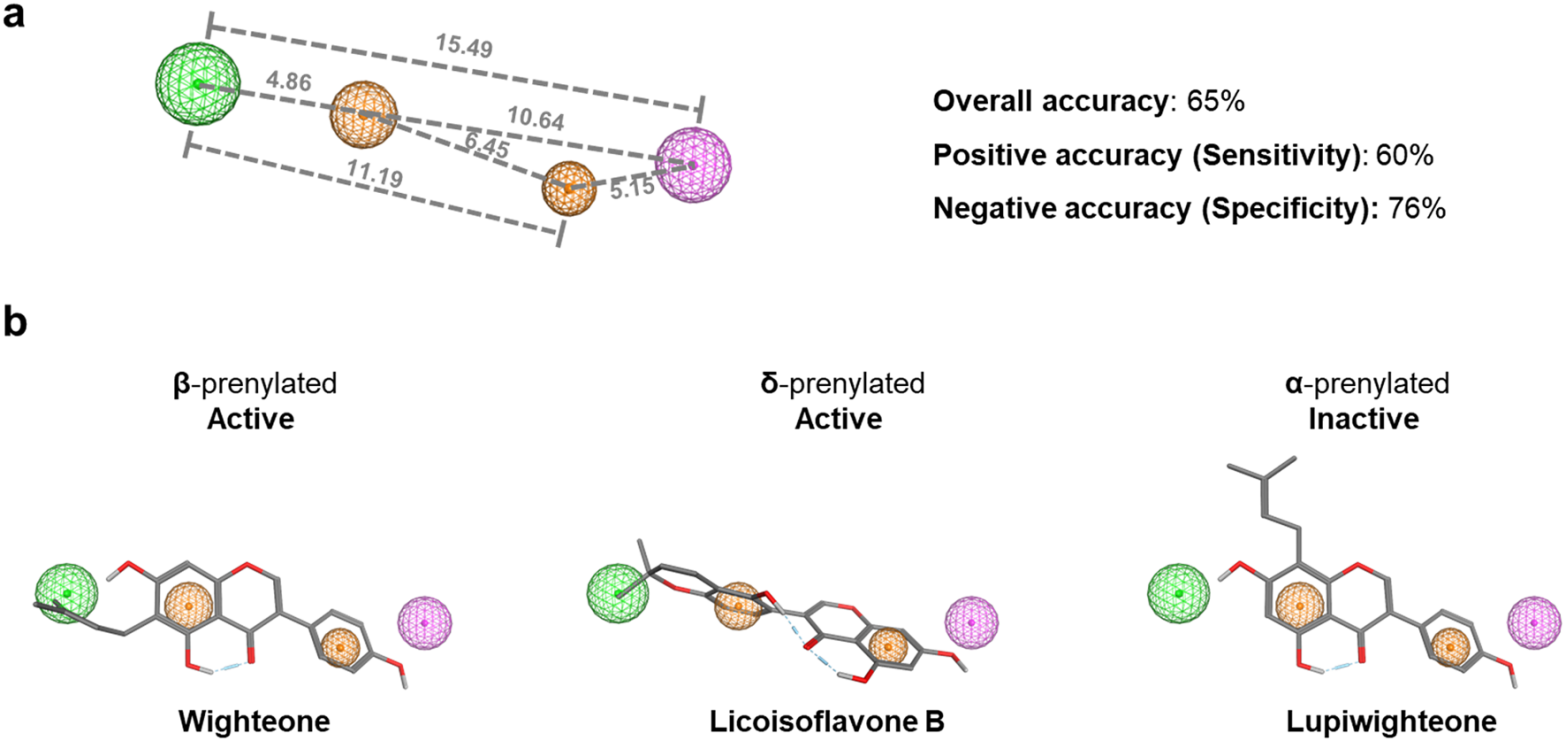
Ligand-based pharmacophore model for active anti-MRSA (MIC ≤ 25 μg/mL) prenylated (iso)flavonoids. The colour of the spheres represents the following features: orange spheres represent aromatic rings, the green and the pink spheres represent hydrophobic features and hydrogen bond donor projections (i.e. features that denote the presence of possible hydrogen bond partners), respectively. Numbers represent the distance between the features in Ångström. The donor/acceptor projections have a radius of 1.2 Å, the aromatic features have a radius of 0.8 Å and 1.0 Å, left and right respectively and the hydrophobic feature has a radius of 1.2 Å. Percentages correspond to the different prediction accuracies (overall, positive and negative) of the model (**a**). The fitting of molecules in the pharmacophore with respect to their potency are also illustrated (**b**).

To understand further the effect of prenylation and of other substituents on the antibacterial activity against MRSA, a ligand-based pharmacophore model was developed. The analysis of the fit of the compounds in the 3D-pharmacophore model revealed four structural features mapping the active molecules; two aromatic ring features (orange) corresponding to the A- and B-ring of (iso)flavonoids, one hydrophobic feature representing the prenyl group (green) and a hydrogen donor projection feature referring to the position of a potential hydrogen bond partner (pink) (Fig. 4a). The pharmacophore model constructed for MRSA had a 65% overall accuracy (sum of molecules predicted correctly), a 60% positive accuracy (active molecules predicted correctly) and a 76% negative accuracy (inactive molecules predicted correctly). These accuracies are 20% higher than the accuracies derived when using a previously constructed pharmacophore model on *L. monocytogenes*^18^. The hits in pharmacophore search and the quality of fitting of each molecule to the model for MRSA and *L. monocytogenes* are shown in Tables S5 and S6, respectively.

The model predicted correctly all the active prenylated isoflavones, the most abundant subclass of the dataset, 70% of the active isoflavans, 50% of the active pterocarpans and 43% of the active pterocarpenes. Figure 4b illustrates examples of isoflavones prenylated at different positions with respect to their fitting quality into the pharmacophore model. Prenylated isoflavones on the β-position (A-ring) and δ-position (B-ring) of the isoflavonoid backbone map all the essential pharmacophore features. In contrast, α-prenylation failed to map the hydrophobic moiety (A-ring).

However, the model failed to predict the active di-prenylated isoflavanones (only the di-prenylated members of this subclass were active), while it correctly predicted analogue molecules such as di-prenylated isoflavones and di-prenylated isoflavans. The simultaneous absence of a double bond in the C-ring and the presence of a carbonyl group in isoflavanones, contrary to isoflavones and isoflavans, respectively, seems to significantly affect the orientation of the B-ring (Fig. S8). This different B-ring orientation could be the reason for the lack of fit of isoflavanones in the pharmacophore. Five molecules (**8**, **25**, **29**, **72** and **77, **Table S1) were found to be false positives during the pharmacophore search (Fig. S9); These molecules are typical examples of activity cliffs, i.e. structurally similar molecules with large difference in potency^54^.

## Discussion

### Hydrophobicity and electrostatic interactions are the main determinants for the anti-MRSA activity of prenylated (iso)flavonoids

Traditionally, the antimicrobial potency of prenylated (iso)flavonoids was attributed to their increased hydrophobicity mainly due to the presence of the prenyl group^11,55^. Generally, the amphiphilic cytoplasmic membrane is hypothesized as the first target of potential antibacterials^56^. Based on the ability of other antibiotics (of similar or larger size than prenylated (iso)flavonoids) to penetrate the bacterial cell wall, it is expected that prenylated (iso)flavonoids can also cross the open structure of the cell wall^57^ and access the membrane^58^. Nonetheless, possible interactions of prenylated (iso)flavonoids with the cell wall cannot be completely excluded.

**Figure 5:**
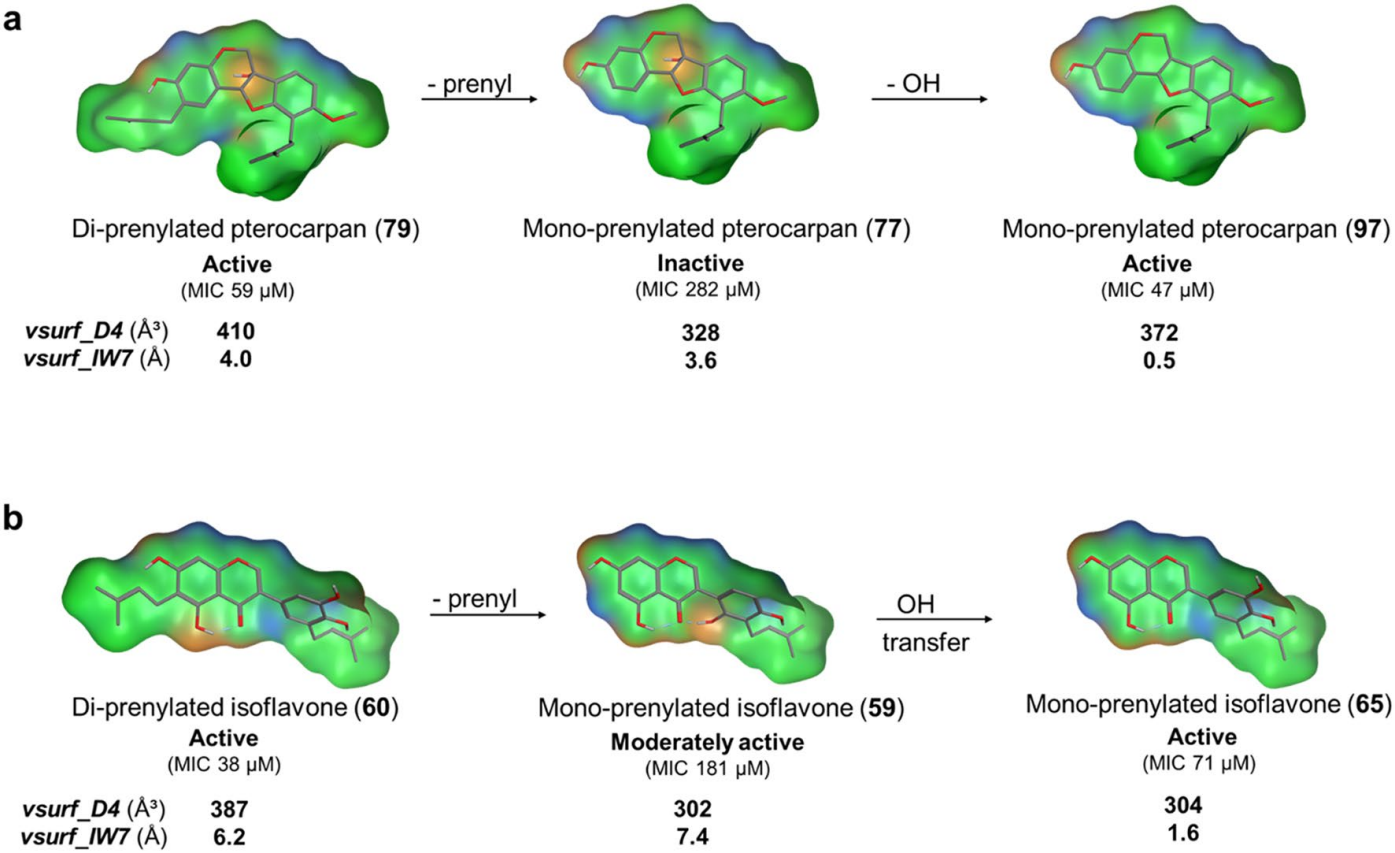
Surface maps of a series of active, moderately active and inactive prenylated pterocarpans (**a**) and isoflavones (**b**). Isoflavonoids from the same subclass (i.e. **a** or **b**) are analogues, differing only in the presence, absence or relocation of one functional group (indicated by the arrows). These structural differences influence their hydrophobic volume (*vsurf_D4,* Å^3^) and the distribution of the hydrophilic regions (*vsurf_IW7,* Å) and therefore the anti-MRSA activity. Mildly polar regions are coloured in blue, H-bonding in orange and hydrophobic regions in green.

In this study, continuous hydrophobicity with balanced hydrophilic groups in the form of the descriptors *vsurf_IW7*, *vsurf_D4* and *vsurf_CW3*, were the most strongly correlated properties to anti-MRSA activity, in accordance with previous results against the Gram-positive *L. monocytogenes*^18^. The contribution of the hydrophobic volume (*vsurf_D4*) and hydrophilic integy moment (*vsurf_IW7*) to the anti-MRSA activity is illustrated in Fig. 5. Di-prenylated (iso)flavonoids are characterized by an extensive hydrophobic volume, as in molecules (**79**) and (**60**). In most cases, extensive hydrophobic volume accounts for the increased antimicrobial potency of the molecules^18^. Decreasing their hydrophobic volume by removal of one prenyl-group, as in molecules (**77**) and (**59**) indeed decreased the anti-MRSA activity. However, further removal of the *C*6a hydroxyl-group (molecule (**77**) to (**97**)) (Fig. 5a) restored the hydrophobic volume to a certain extent, reduced the hydrophilic moments in the molecule (*vsurf_IW7*), leading to higher anti-MRSA activity.

Interestingly, transferring the OH group from *C*5′ to *C*6′ (molecule (**59**) to (**65**)) (Fig. 5b) restored the balance between the hydrophilic moments in the molecule, enhancing the anti-MRSA potency, even without influencing the hydrophobic volume. In this respect, isolupalbigenin (**62**) together with erybraedin A (**78**) and eryvarin W (**106**) were highlighted as the most active (di-prenylated) compounds of the dataset (“Extended prenylated (iso)flavonoid dataset for QSAR modelling of anti-MRSA activity” section). Isolupalbigenin (**62**) has relatively unfavorable distribution of hydrophilic groups (*vsurf_IW7* = 4.5 Å) compared to the other two molecules (Fig. S3), both having *vsurf_IW7* = 0.0 Å). By changing the position of the OH group from the *C*5 of the A-ring to the *C*6’ of the B-ring (Fig. S3), the new molecule has optimal distribution of the hydrophilic groups (*vsurf_IW7* = 0.0 Å) and was predicted to have the highest activity from all molecules of the dataset.

From the above it becomes also evident that prenylated (iso)flavonoids scoring low in hydrophobicity can still be active anti-MRSA agents (e.g. molecule **65, **Fig. 5b). This observation agrees with the findings of our pharmacophore model, where the importance of single, and not necessarily double, prenylation together with a hydrogen bond acceptor were highlighted. Charge was highlighted as the second most important molecular property for the antibacterial activity of prenylated (iso)flavonoids. Large positive partial charges (*PEOE_VSA_PPOS* and *PEOE_VSA* + *2*) and formal negative charges (*h_pavgQ*) suggest the importance of electrostatic interactions^49^. Sadgrove et al*.* (2020) also associated the importance of electrostatic interactions such as hydrogen-bonding with anti-(MR)SA activity^21^.

### New insights in the possible interaction of active anti-MRSA agents with the cytoplasmic membrane

**Figure 6:**
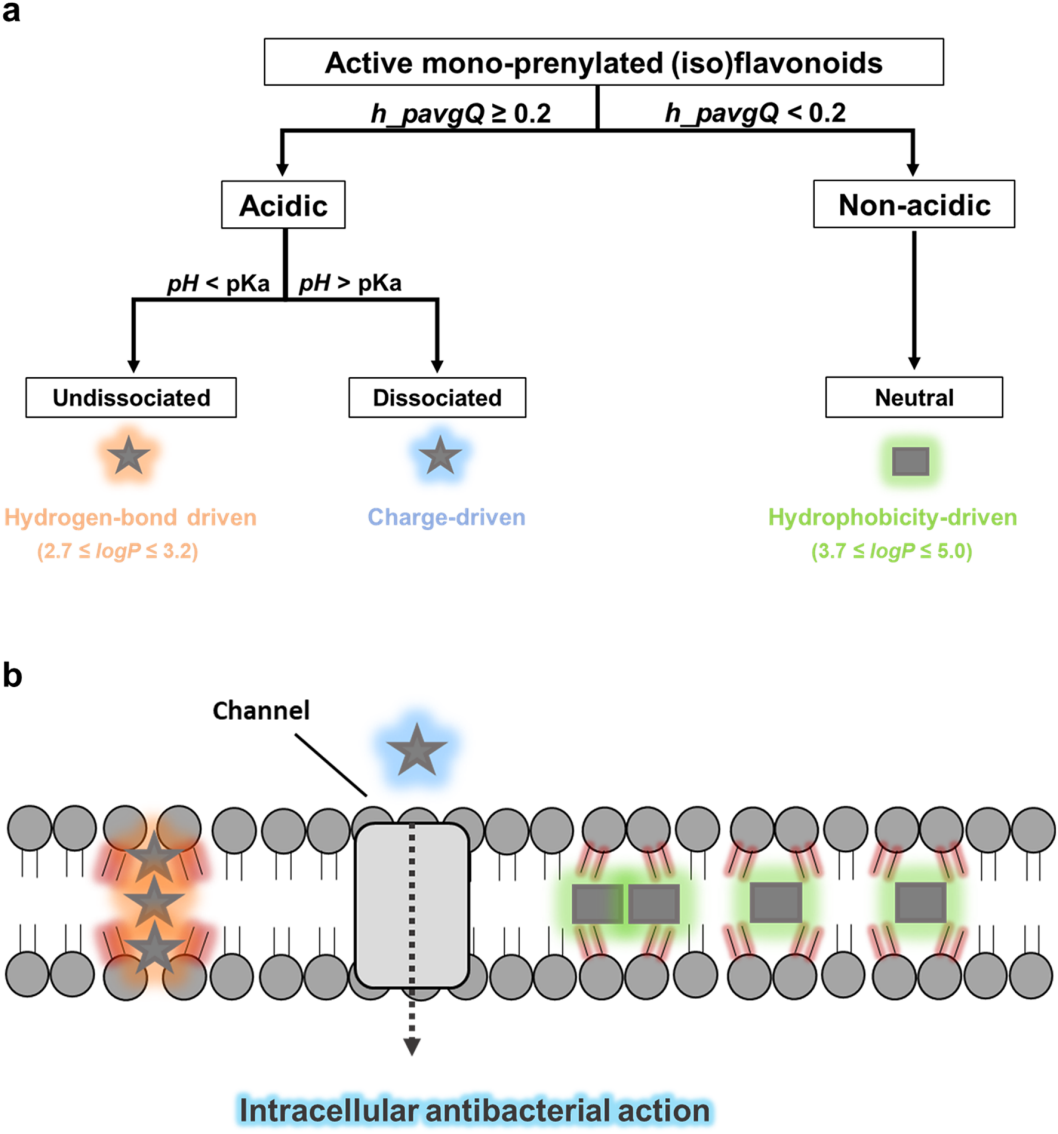
Proposed classification of active (MIC ≤ 25 µg/mL) prenylated (iso)flavonoids based on their potential to be negatively charged at pH 7. Molecules with high potential to be dissociated at pH 7 (*h_pavgQ* ≥ 0.2), i.e. acidic, were considered for their dissociated and undissociated forms separately. Molecules that remain fully undissociated at pH 7 (*h_pavgQ* < 0.2), i.e. non-acidic, were considered neutral (**a**). Hypothesized interactions of active anti-MRSA prenylated (iso)flavonoids with the cytoplasmic membrane; possible interactions of prenylated (iso)flavonoids with the cell wall are not considered in this study (**b**). Stars represent active acidic mono-prenylated (iso)flavonoids, whereas rectangular shapes represent active, neutral mono-prenylated (iso)flavonoids. Different glows around the shapes indicate hypothesized, predominant driving forces that mediate cellular uptake or interaction with the membrane; blue denotes a negative charge, orange indicates strong hydrogen bonding and green glow represents hydrophobicity-driven interaction with the lipid bilayer. The intensity of red shading around phospholipids shows the level of compromise of membrane integrity.

Based on the main molecular properties highlighted by the QSAR study and the pharmacophore model, it is hypothesized that different active mono-prenylated anti-MRSA (iso)flavonoids might be differently taken up by the cell or interact with the membrane. Three ways of interaction of prenylated (iso)flavonoids with the cytoplasmic membrane are proposed (Fig. 6).

#### Acidic prenylated isoflavonoids might interact with both the membrane and with intracellular targets

First, prenylated (iso)flavonoids with high potential to be charged at pH 7 are hypothesized to interact differently from the ones that remain neutral across the pH range. The average total formal charge sum across protonation states at pH 7^47^ is represented by the descriptor, *h_pavgQ* (Table S2)*.* Twenty percent of the active mono-prenylated (iso)flavonoids had an absolute *h_pavgQ* value higher than 0.2. These molecules were considered acidic and both their dissociated and undissociated form were taken into account (Fig. 6a, left side). The subclass of isoflavones is the predominant representative subclass (83%) of acidic, active mono-prenylated (iso)flavonoids. All, but one, molecules of this category in our dataset are characterized by the presence of three or more free hydroxyl groups. Prenylated (iso)flavones have been shown experimentally to permeabilize the membrane of MRSA and other Gram-positive bacteria^18,59,60^. Yet, the exact mechanism of membrane permeabilization has not been thoroughly investigated.

Recently, Li et al*.* (2018) showed that a very active (MIC 1 μg/mL, 2 μM) di-prenylated xanthone (alpha-mangostin) forms membrane-spanning, intermolecular aggregates primarily through hydrogen bonding^58^. This was shown through molecular dynamic simulation experiments. Aggregate formation ultimately led to membrane destabilization and subsequent water translocation across the membrane, without pore formation^58^. This is in line with the membrane permeabilization of Gram-positive bacteria observed by prenylated isoflavones^18,60^. Interestingly, it was specifically highlighted that the presence of three hydroxyl groups in alpha-mangostin are crucial for transmembrane aggregate formation, while their absence would make alpha-mangostin soluble in the lipid bilayer^58^. The formation of intermolecular hydrogen bonds possibly facilitates the unfavourable presence of polar groups into the hydrophobic interior of the membrane, stabilizing the cluster. Alpha-mangostin is structurally similar to the active isoflavones in our dataset, e.g. a mono-prenylated (wighteone, **74**) and a di-prenylated one (lupalbigenin, **67**), although the xanthone is more planar^61^ (Fig. S10). The hydrogen bond strengths of mangostin and the two analogue isoflavones were quantified (*h_ema* and *h_emd* descriptors, Table S2, Molecular Operating Environment, MOE). The sum of hydrogen bond donor (HBD) and acceptor (HBA) strengths for these molecules were comparable (HBD: 10.2 kcal/mol for wighteone, 10.9 kcal/mol for lupalbigenin and 11.4 kcal/mol for mangostin; HBA: 4.6 kcal/mol for the two prenylated isoflavones and 5.6 kcal/mol for the xanthone). Since aggregate formation by the di-prenylated xanthone was shown to be primarily hydrogen-bond driven, possibly this mechanism of action is also employed by prenylated isoflavones given the similar degree of hydrogen-bond capacity.

Xanthone transmembrane aggregates were formed after a high enough concentration of the molecule reaches the lipid tail region of the membrane. Although wighteone (**74**) and lupalbigenin (**67**) have similar hydrogen-bond capacities, the presence of only one prenyl group in the former should increase the free energy barrier of membrane penetration compared to the latter^41^. Possibly, higher concentrations are needed for mono-prenylated isoflavones to form these aggregates than for their di-prenylated counterparts, something which is also reflected in the higher MICs observed for the former.

The mechanism of membrane activity proposed by Li et al*.* (2018) considered only the undissociated form of the molecule^58^. A negative charge in the dissociated acidic isoflavones would be repelled by the polar (phosphate) head groups of the membrane and would not allow membrane penetration^58,62^. Instead, the dissociated acidic isoflavones might be transported to the cytosol through transmembrane carrier proteins or by active transport^63,64^ (Fig. 6b, blue star) similar to what has been shown for other anionic antibiotics, such as carbenicillin and quinolones^65^. The proposed membrane transportation of these molecules implies that the membrane is not the only target for their anti-MRSA activity and their mode of action is further complemented by activity inside the cytosol. Recently, a di-prenylated flavone was shown to inhibit the biosynthesis of phosphatidic acids in the cytosol, the repair mechanism of bacterial membranes^59^ after membrane disruption. Noticeable inhibition (> 99%) of MRSA was observed within 2 h of exposure to this prenylated flavonoid^59^, similar to what was found in Fig. 1.

It is therefore proposed that the dissociated and undissociated forms of antibacterial acidic (iso)flavonoids might employ different modes of action (Fig. 6b, orange and blue stars). Internalization of antibacterials in different ways depending on their protonation state has been shown before for Gram-negatives^66^.

#### *Non-acidic prenylated isoflavonoids might be internalized by the membrane *via* diffusion*

Hydrogen-bonding, formal charge and hydrophobicity were the main molecular properties determining anti-MRSA activity. It was hypothesized that acidic prenylated isoflavonoids may interact with both the membrane and with intracellular targets, depending on their protonation state. Nevertheless, 80% of the active mono-prenylated isoflavonoids had an absolute *h_pavgQ* value of less than 0.2, meaning that these molecules remain neutral at the pH of the medium. This category of non-acidic prenylated (iso)flavonoids (Fig. 6a, right side) comprised compounds with less than 3 hydroxyl groups and higher *logP* values (ranging from 3.7 to 5.0) than the acidic prenylated isoflavonoids (*logP* ≤ 3.2 for the undissociated species). It is therefore hypothesized that hydrophobicity becomes the main driver for interaction of these molecules with the membrane^58^. Nevertheless, the presence of a few (< 3) hydroxyl groups in these molecules, may also trigger aggregate formation, similarly to the acidic, undissociated (iso)flavonoids. Individually or as aggregates, these molecules might be soluble within the lipid bilayer^58^, due to their higher hydrophobicity, ultimately disturbing its integrity^65^ (Fig. 6b, green rectangulars). Contrary to prenylated isoflavones (the acidic group in Fig. 6), a few examples from the non-acidic subclasses were shown not to permeabilize the cytoplasmic membrane, but have been hypothesized to still disrupt its integrity by other means^18^.

Interestingly, neutral prenylated isoflavonoids can be planar (pterocarpenes) or non-planar (pterocarpans and isoflavans), while acidic isoflavonoids included in this study are all non-planar. Notably, the two most active mono-prenylated isoflavonoids studied were the pterocarpan Orientanol B (**93**) (six times more active than its pterocarpene analogue, Dehydroglyceollin IV (**102**), Table S1 and Fig. S2)) and the isoflavan 4′-*O*-methyl-glabridin (**17**). Both molecules adopt non-planar conformations (Fig. S8). Planarity has been associated to the extent of insertion in the cytoplasmic membrane and the level of disruption of membrane integrity^66–68^. It has been hypothesized that non-planar molecules disrupt membrane integrity more effectively due to better interaction acyl chains^58,67,68^, possibly leading to higher antimicrobial activity. In contrast, planar, molecules may interact less efficiently with the acyl chains, despite their rapid diffusion into the membrane.

Overall, the in silico models developed in the study showed for the first time that active prenylated (iso)flavonoids can have fundamentally different molecular properties (such as acidic or highly hydrophobic), suggesting potentially different modes of action. Prenylated (iso)flavonoids might employ different mechanisms for cell uptake or interaction with the cytoplasmic membrane. Information on the contribution of these molecular properties to the anti-MRSA activity can aid the design and development of novel antibacterial agents against Gram-positive bacteria. The best QSAR model developed in the study can be used as a screening tool to predict the (level of) activity of new antibacterial agents against Gram-positive bacteria.

## Supplementary Information

Supplementary Information.
