# Author Correction: Insights into the molecular properties underlying antibacterial activity of prenylated (iso)flavonoids against MRSA

**DOI:** 10.1038/s41598-022-07664-9

**Published:** 2022-03-02

**Authors:** Sylvia Kalli, Carla Araya‑Cloutier, Jos Hageman, Jean‑Paul Vincken

**Affiliations:** 1 Laboratory of Food Chemistry, Wageningen University & Research, Wageningen, The Netherlands; 2 Biometris, Applied Statistics, Wageningen University & Research, Wageningen, The Netherlands

Correction to: *Scientific reports* 10.1038/s41598-021-92964-9, published online 09 July 2021

The Article contained an error in Table 2, where the “Equation” value was incorrect for “n4”. The incorrect and correct value appears below.

Incorrect:

**Table:** 

n	Equation
4	y = 2.551(0.343) − 0.178(0.045)*h_emd_C + 0.006(0.001)*vsurf _D4 − 0.050(0.017)*vsurf _IW7 − 0.047(0.016)*E_vdw

Correct:

**Table:** 

n	Equation
4	y = 2.551(0.343) − 0.178(0.045)*h_emd_C + 0.006(0.001)*vsurf _D4 − 0.050(0.017)*vsurf _IW7 + 0.047(0.016)*E_vdw

The original Article has been corrected.

