# Supplementary material for: Insights into the molecular properties underlying antibacterial activity of prenylated (iso)flavonoids against MRSA
Source: Sci Rep. 2021 Jul 9;11:14180. doi: 10.1038/s41598-021-92964-9 (PMC8270993; doi:10.1038/s41598-021-92964-9)
Supplement: Supplementary file 1 — Supplementary Information. [file 41598_2021_92964_MOESM1_ESM.pdf]

Supplementary files 'Insights into the molecular properties underlying antibacterial activity of prenylated (iso)flavonoids against MRSA' by Kalli, S., Araya-Cloutier, C., Hageman, J. and Vincken, J.-P.

Figures

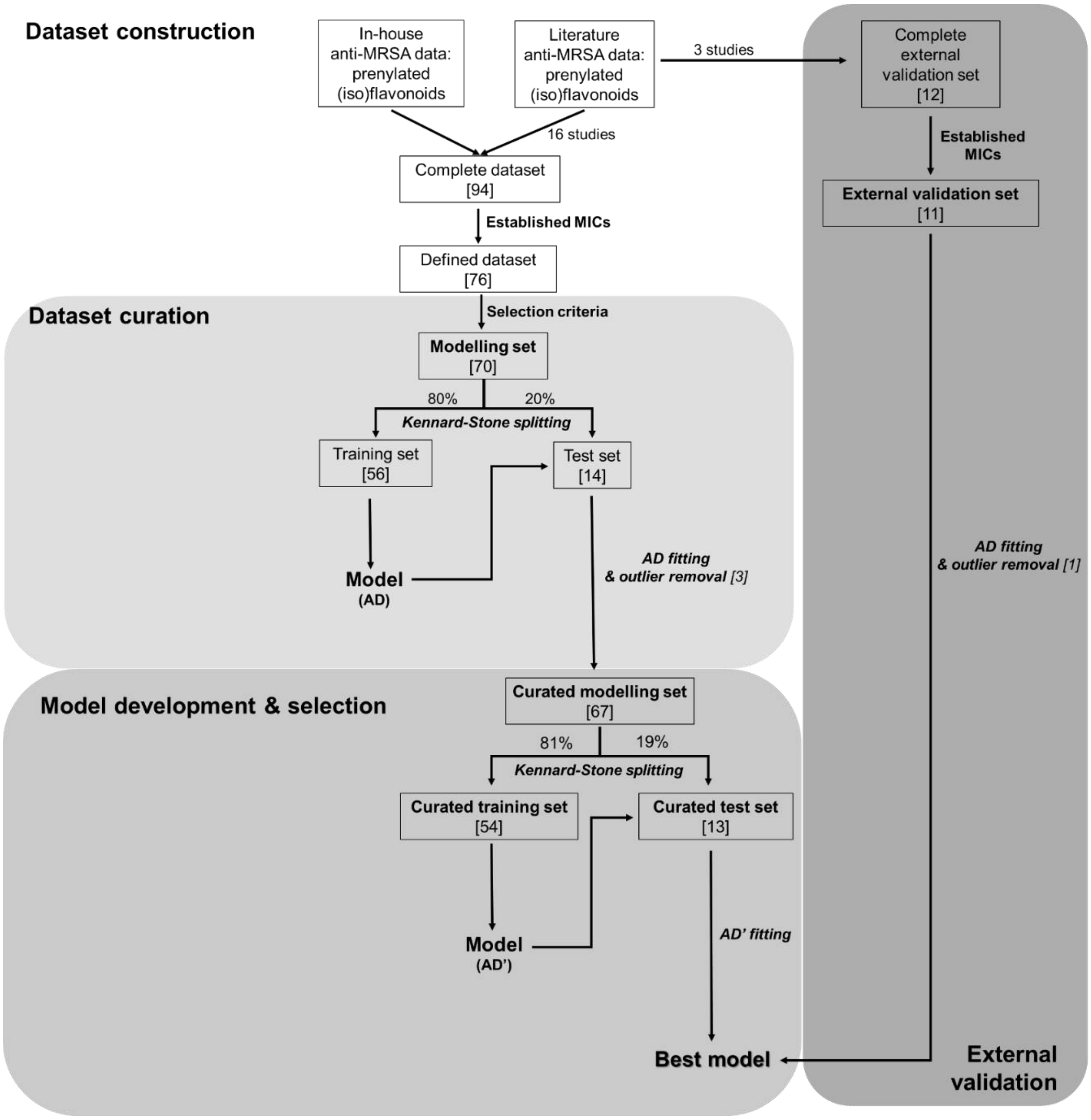

Figure S1. Outline of approach followed for QSAR modelling; AD: applicability domain; Numbers in brackets refer to the number of compounds comprised in each set. Colour intensity of the shadings shows the sequence of steps followed for model development, selection and external validation.

Supplementary files 'Insights into the molecular properties underlying antibacterial activity of prenylated (iso)flavonoids against MRSA' by Kalli, S., Araya-Cloutier, C., Hageman, J. and Vincken, J.-P.

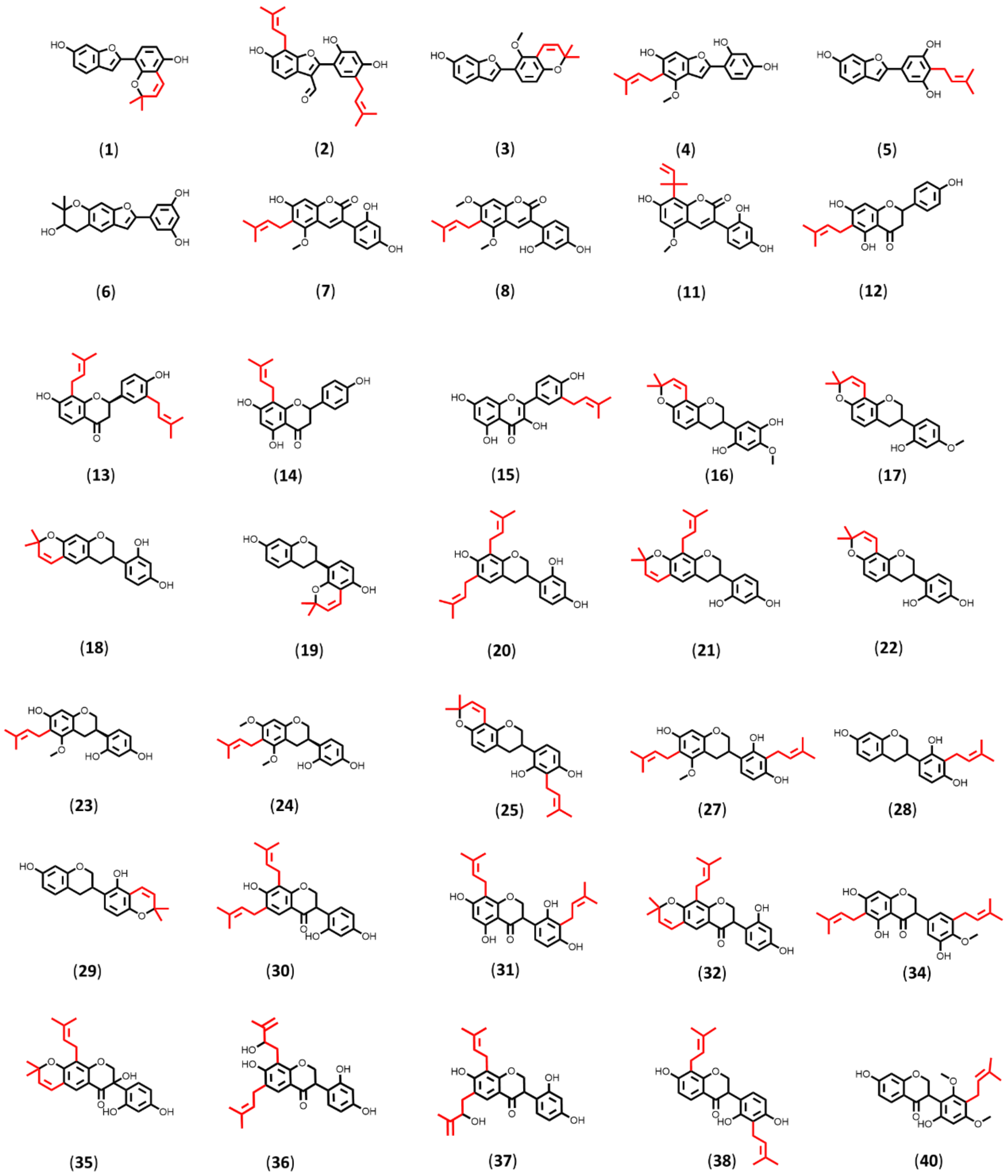

Figure S2. Structures of prenylated (iso)flavonoids with established MICs from **Table S1**.

Supplementary files 'Insights into the molecular properties underlying antibacterial activity of prenylated (iso)flavonoids against MRSA' by Kalli, S., Araya-Cloutier, C., Hageman, J. and Vincken, J.-P.

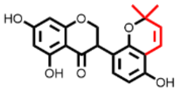
(41)

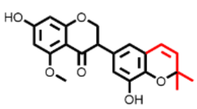
(42)

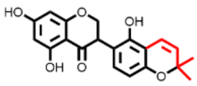
(43)

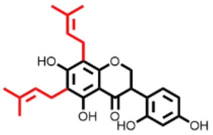
(44)

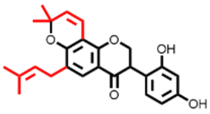
(45)

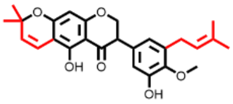
(46)

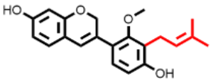
(47)

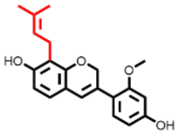
(48)

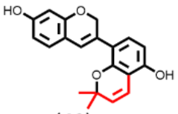
(49)

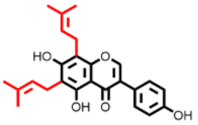
(50)

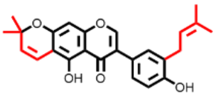
(52)

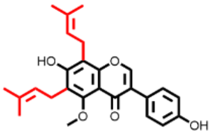
(53)

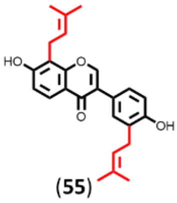
(55)

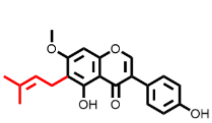
(56)

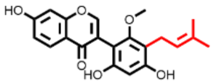
(57)

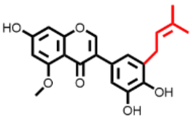
(58)

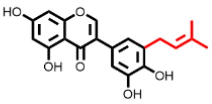
(59)

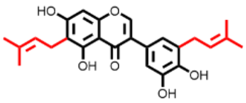
(60)

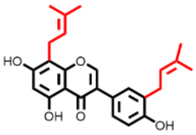
(62)

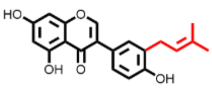
(64)

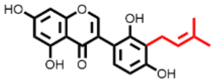
(65)

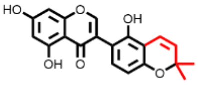
(66)

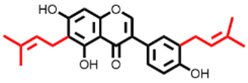
(67)

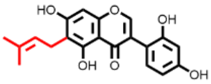
(69)

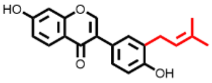
(70)

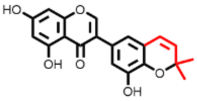
(72)

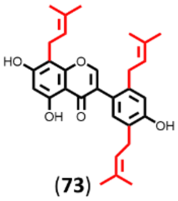
(73)

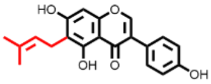
(74)

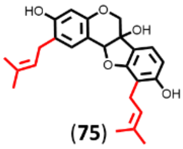
(75)

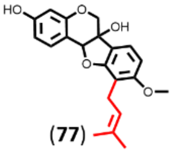
(77)

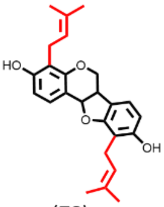
(78)

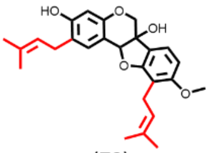
(79)

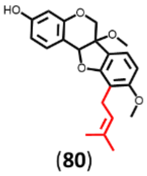
(80)

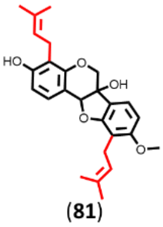
(81)

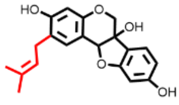
(86)

Figure S2. Continued

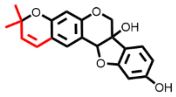

(87)

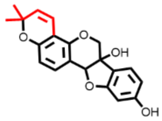

(88)

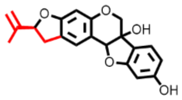

(89)

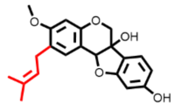

(90)

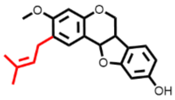

(93)

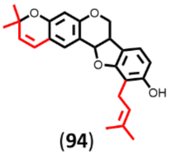

(94)

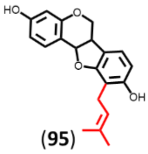

(95)

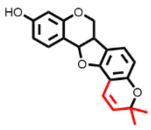

(96)

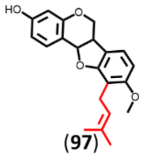

(97)

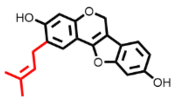

(98)

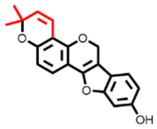

(99)

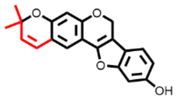

(100)

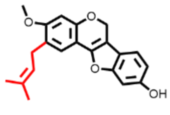

(102)

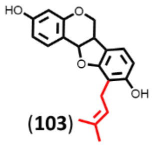

(103)

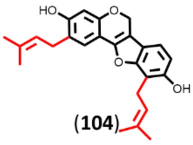

(104)

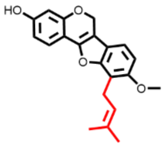

(105)

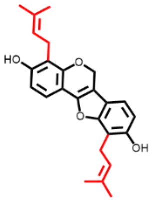

(106)

Figure S2. Continued

Supplementary files 'Insights into the molecular properties underlying antibacterial activity of prenylated (iso)flavonoids against MRSA' by Kalli, S., Araya-Cloutier, C., Hageman, J. and Vincken, J.-P.

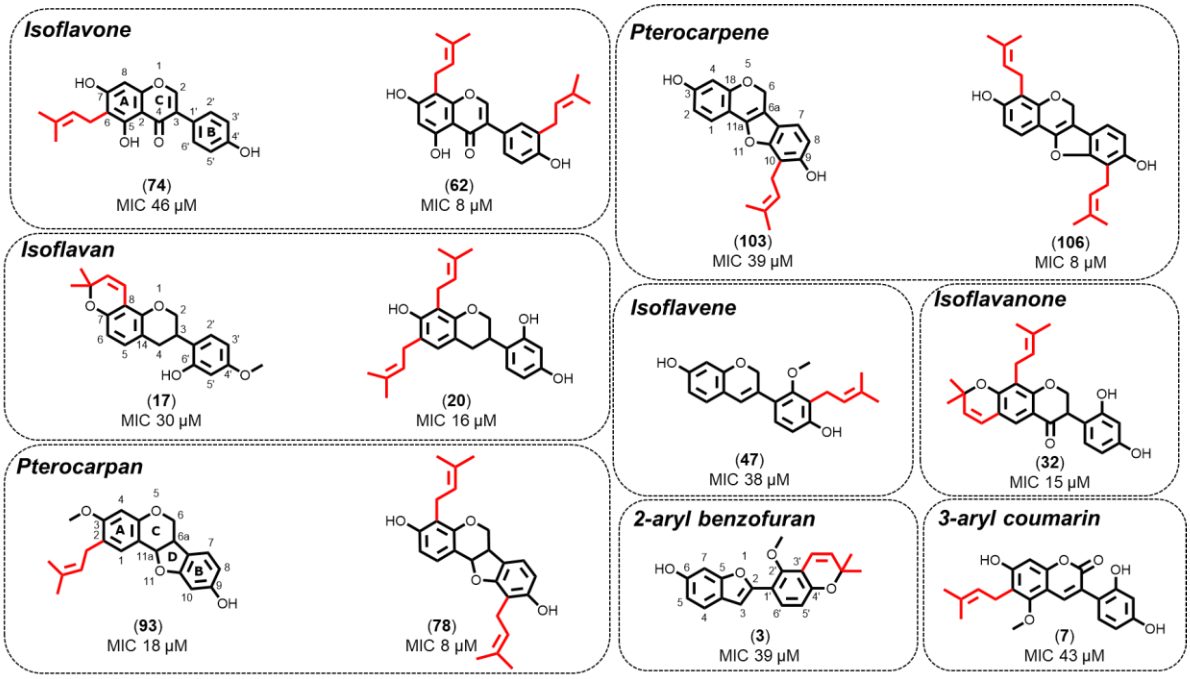

Figure S3. Structures of representative active (MIC ≤ 25 µg/mL, 8-64 µM) mono- and di-prenylated isoflavonoids against MRSA per subclass. Only mono-prenylated derivatives of isoflavenes, 2-aryl benzofurans and 3-aryl coumarins were present in the curated modelling and external validation sets (**Table S1**), whereas only di-prenylated isoflavanones were active anti-MRSA agents. The prenyl groups are highlighted in red.

Supplementary files 'Insights into the molecular properties underlying antibacterial activity of prenylated (iso)flavonoids against MRSA' by Kalli, S., Araya-Cloutier, C., Hageman, J. and Vincken, J.-P.

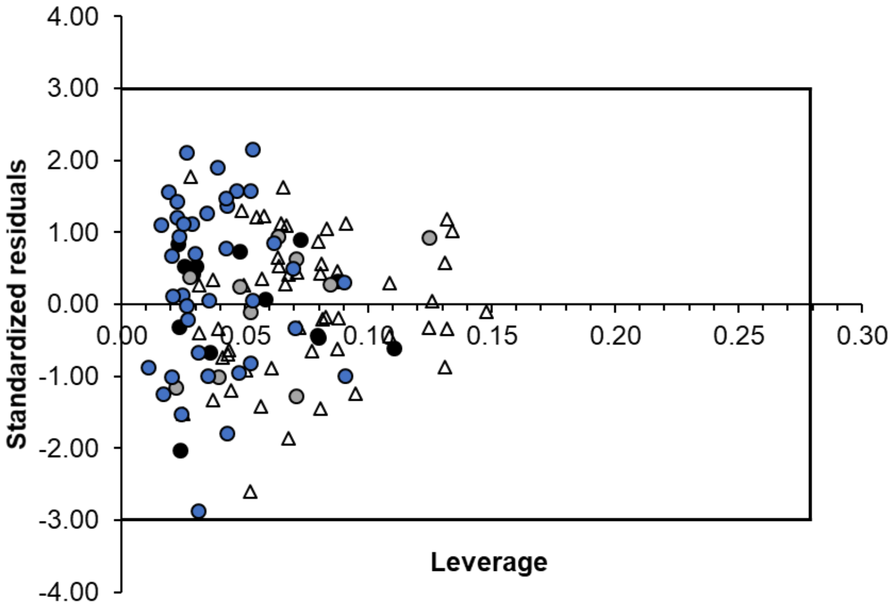

Figure S4. Applicability domain of the selected best model for MRSA. The location of molecules from the training (triangles), test (black circles), external validation (grey circles) sets and the set of compounds tested against other Gram-positives (blue circles) are depicted [1].

Supplementary files ‘Insights into the molecular properties underlying antibacterial activity of prenylated (iso)flavonoids against MRSA’ by Kalli, S., Araya-Cloutier, C., Hageman, J. and Vincken, J.-P.

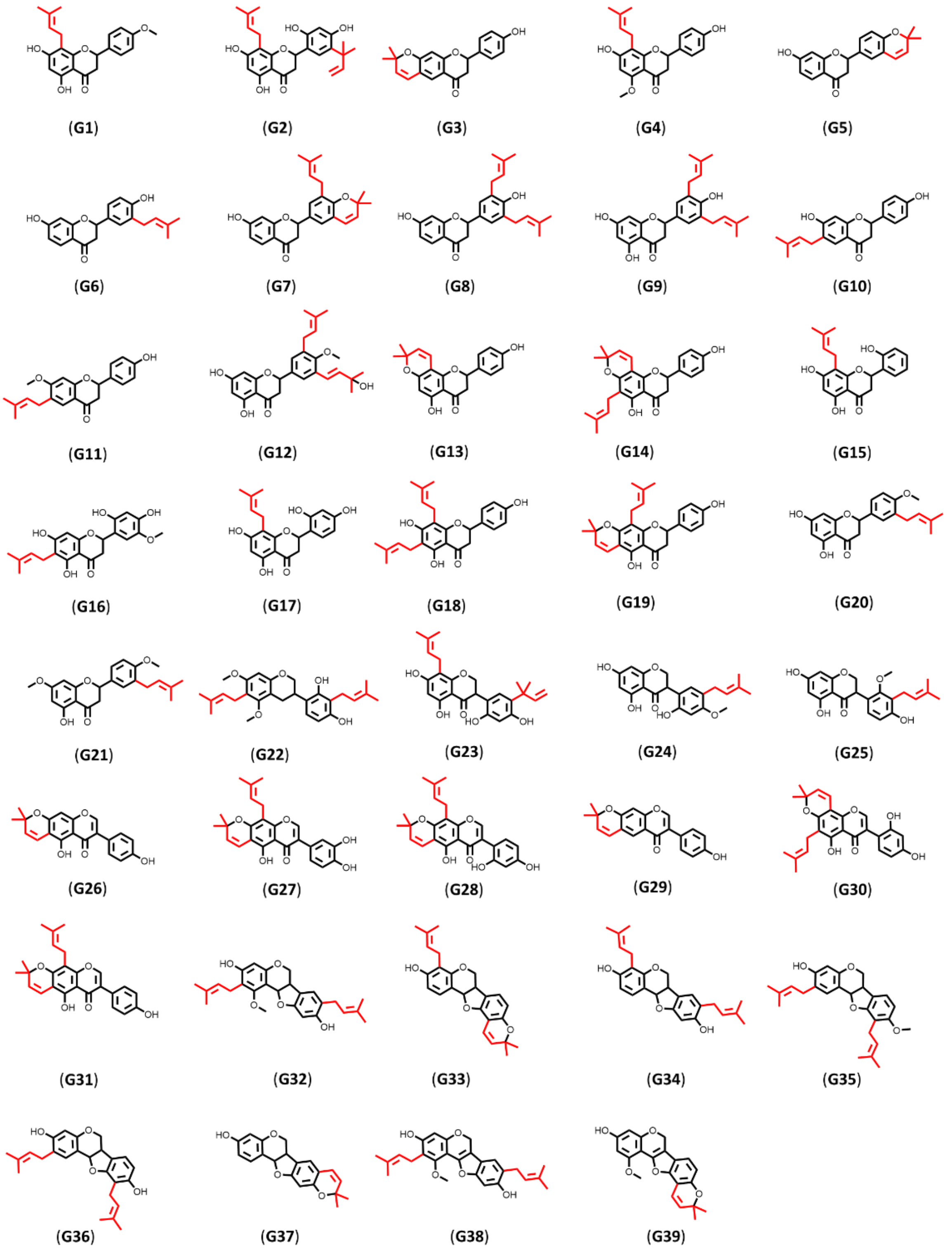

Figure S5. Structures of prenylated (iso)flavonoids tested against other Gram-positives (except MRSA) and used to assess the applicability of the best QSAR model developed on MRSA. The structures of the molecules that have also been tested against MRSA can be found in **Figure S2**.

Supplementary files 'Insights into the molecular properties underlying antibacterial activity of prenylated (iso)flavonoids against MRSA' by Kalli, S., Araya-Cloutier, C., Hageman, J. and Vincken, J.-P.

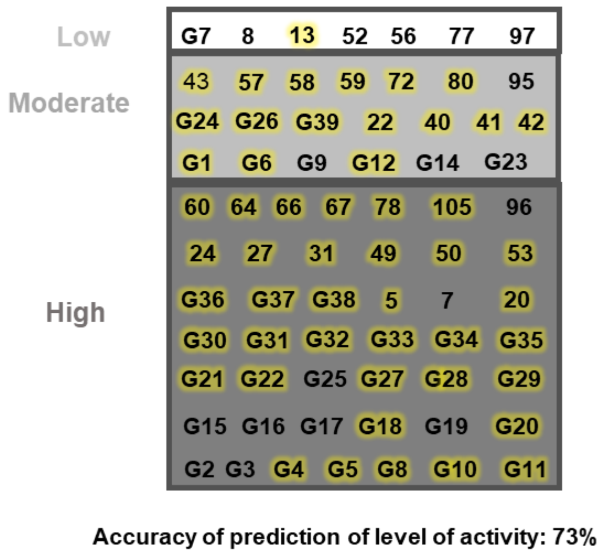

Figure S6. Prediction of level of antibacterial activity, ie. low (MIC ≥ 100 µg/mL , moderate (25 < MIC < 100 µg/mL) and high (MIC ≤ 25 µg/mL) of prenylated (iso)flavonoids against other Gram-positive bacteria by using the best QSAR model obtained for MRSA. Numbers refer to the prenylated (iso)flavonoids of **Table S3**; "G" in front of the numbers refer to molecules which have not been tested against MRSA. A yellow glow around the numbers demonstrates that their level of activity was predicted correctly. Compounds for which the predicted activity differed by one two-fold dilution from their experimentally determined one were still considered as correctly predicted [2,3].

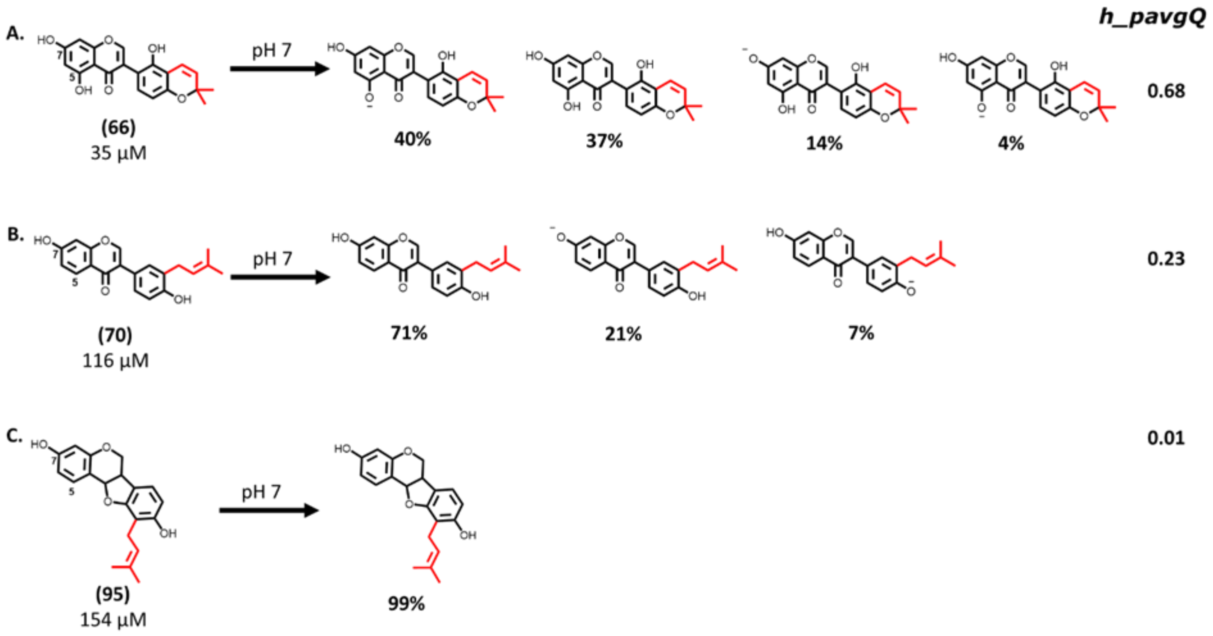

Figure S7. Protonation states and their relative abundances at pH 7 of different prenylated isoflavonoids with respect to their anti-MRSA activity and the descriptor, *h_pavgQ*.

Supplementary files 'Insights into the molecular properties underlying antibacterial activity of prenylated (iso)flavonoids against MRSA' by Kalli, S., Araya-Cloutier, C., Hageman, J. and Vincken, J.-P.

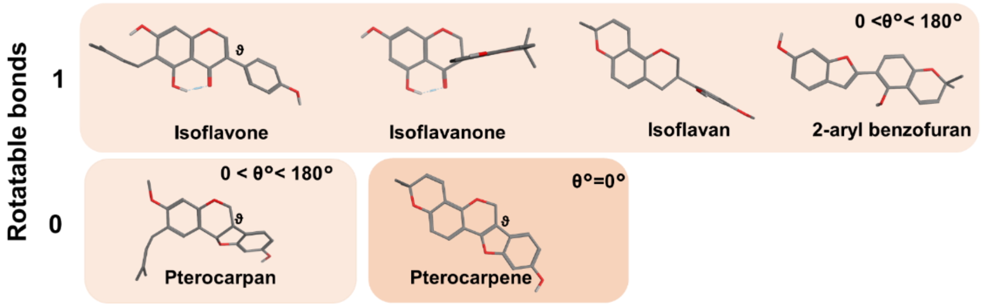

Figure S8. Examples of energy minimized structures of mono-prenylated isoflavonoids from selected subclasses. Rotatable bonds refer to the backbone and ϑ denotes the dihedral bond.

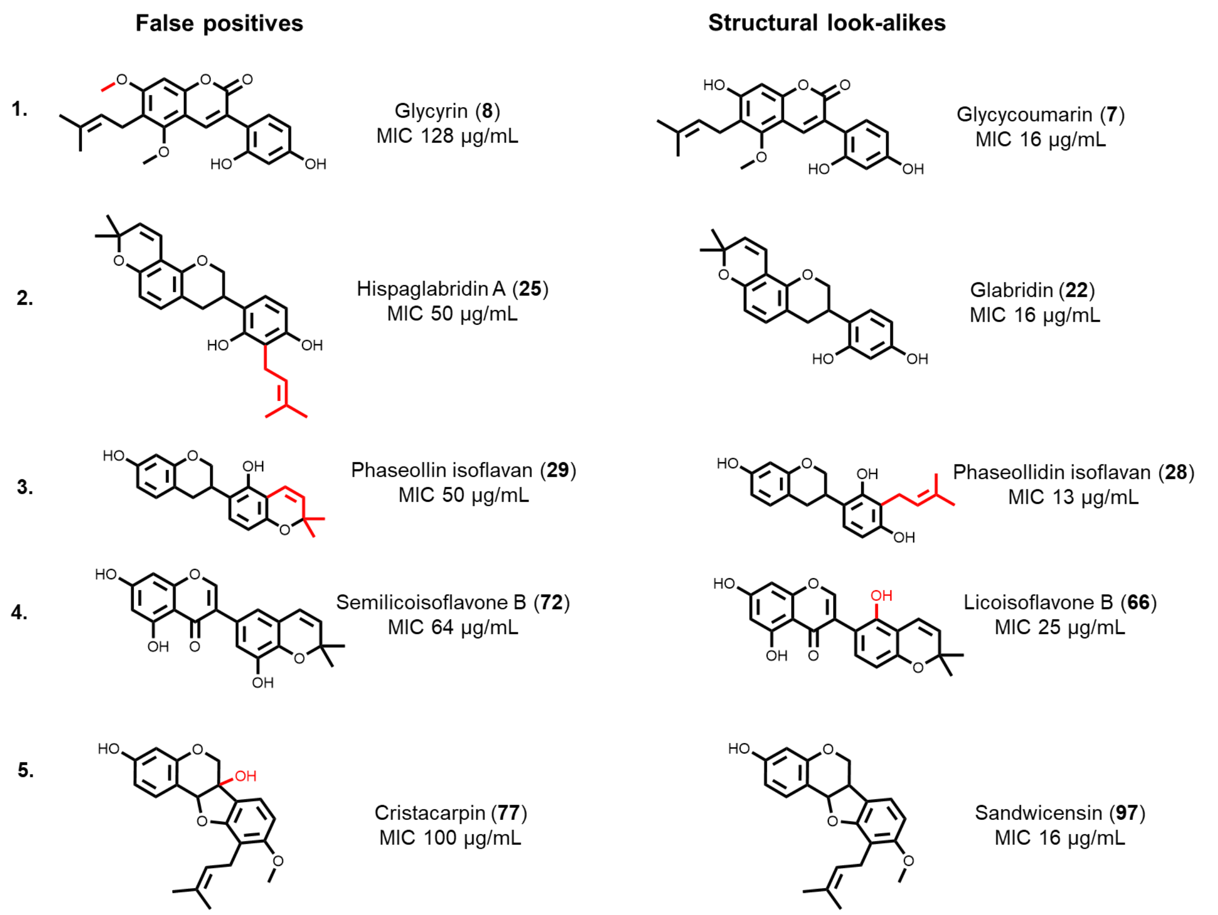

Figure S9. False positives (left) in the pharmacophore search. These molecules are "activity cliffs" in the database. Their structural look-alikes are presented on the right and the structural differences that result in substantial change in activity are presented in red.

Supplementary files ‘Insights into the molecular properties underlying antibacterial activity of prenylated (iso)flavonoids against MRSA’ by Kalli, S., Araya-Cloutier, C., Hageman, J. and Vincken, J.-P.

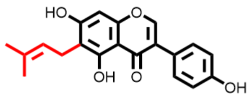

Wighteone (isoflavone)

MIC 16 μg/mL (46 μM)

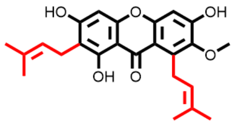

Alpha-mangostin (xanthone)

MIC 1 μg/mL (2 μM)

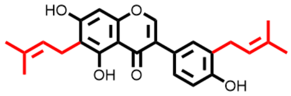

Lupalbigenin (isoflavone)

MIC 4 μg/mL (10 μM)

Figure S10. Structure and anti-MRSA activity of alpha-mangostin [4] in relation to two structurally related prenylated isoflavones, one mono- and one di-prenylated (wighteone and lupalbigenin, respectively).

Supplementary files 'Insights into the molecular properties underlying antibacterial activity of prenylated (iso)flavonoids against MRSA' by Kalli, S., Araya-Cloutier, C., Hageman, J. and Vincken, J.-P.

Tables

Table S1. Anti-MRSA activity of prenylated (iso)flavonoids, experimentally tested and mined from literature. KS stands for the Kennard-Stone splitting, TR stands for the training set, TE for the test set and EV for the external validation set. The % prediction error is defined as ($pMIC_{observed}$-$pMIC_{predicted}$) / $pMIC_{observed}$ *100. Molecules with an asterisk (*) did not comply with the structural criteria (**criterion 1** and **criterion 2** in **Dataset construction**, cr.1 and cr.2) used for and molecules in grey had unestablished minimum inhibitory concentrations (MIC). The structures of compounds with established MICs can be found in **Figure S1**. Di-prenylated (iso)flavonoids are indicated **in bold**. Molecules are sorted based on subclass and ordered alphabetically within each subclass.

ID	(Iso)flavonoid subclass	Name	KS split	MRSA MIC (μg/mL)	MRSA MIC (μM)	% Prediction error	Reference
1	2-arylbenzofuran	Glabrocoumarone A (Kanzonol U)	EV	13	42	3.4	[5]
**2**	**2-arylbenzofuran**	**Eryvarin Q**	*(cr.2)	6	15	4.7	[6]
3	2-arylbenzofuran	Eryvarin U	TR	12.5	39	7.4	[7]
4	2-arylbenzofuran	Licocoumarone	TR	16	47	2.1	[8]
5	2-arylbenzofuran	Moracin C	TR	13	40	0.5	[9]
6	2-arylbenzofuran	Moracin P	*(cr.1)	100	306	10.5	[10]
7	3-arylcoumarin	Glycycoumarin	TR	16	43	8.1	[8]
8	3-arylcoumarin	Glycyrin	TR	128	335	14.4	[8]
9	3-arylcoumarin	Glycyrin permethyl ether	-	>128	n.a.	n.a	[8]
10	3-arylcoumarin	Isorobustone	-	>128	n.a.	n.a	[11]
11	3-arylcoumarin	Licoarylcoumarin	*(cr.1)	32	87	10.2	[8]
12	Flavanone	6-prenylnaringenin	TR	38	110	2.3	This study
**13**	**Flavanone**	**Glabrol**	*	9	24	2.5	This study
14	Flavanone	Sophoraflavanone B	TR	31	92	5.9	[12]
15	Flavonol	Isolicoflavonol	*(cr.2)	25	71	4.9	[10]
16	Isoflavan	3'-OH-4'-O-methylglabridin	TR	16	44	9.9	This study
17	Isoflavan	4'-O-methylglabridin	TR	10	30	3.8	This study
18	Isoflavan	Eryvarin C	TE	25	77	2.2	[13]
19	Isoflavan	Erythbidin A	EV	50	154	8.9	[5]
**20**	**Isoflavan**	**Eryzerin C**	TR	6	16	1.8	[14,15]
**21**	**Isoflavan**	**Eryzerin D**	TR	13	32	3.8	[15]
22	Isoflavan	Glabridin	TR	16	49	2.0	[8]
23	Isoflavan	Glyasperin C	TE	16	45	4.8	[8]
24	Isoflavan	Glyasperin D	TR	16	43	2.7	[8]
**25**	**Isoflavan**	**Hispaglabridin A**	TR	50	127	17.8	This study
26	Isoflavan	Hispaglabridin B	-	>100	n.a.	n.a	This study
**27**	**Isoflavan**	**Licoricidin**	TR	16	38	1.1	[8]
28	Isoflavan	Phaseollidin isoflavan	EV	13	40	0.5	[5]
29	Isoflavan	Phaseollin isoflavan	EV	50	155	8.0	[5]
**30**	**Isoflavanone**	**2,3-Dihydroauriculatin**	EV	25	59	4.2	[16]
**31**	**Isoflavanone**	**3'-dimethylallyl-kievitone**	TR	8	19	3.1	[8]
**32**	**Isoflavanone**	**Bidwillon B**	TR	6.25	15	6.9	[14,17]
33	Isoflavanone	Costarone	-	>50	59	n.a.	[18]
**34**	**Isoflavanone**	**Diprenyl-costarone**	Outlier	6	14	20.8	[18]
**35**	**Isoflavanone**	**Eryvarin V**	TR	25	59	2.6	[19]
**36**	**Isoflavanone**	**Eryvarin Y**	EV	25	59	0.1	[16]

Supplementary files 'Insights into the molecular properties underlying antibacterial activity of prenylated (iso)flavonoids against MRSA' by Kalli, S., Araya-Cloutier, C., Hageman, J. and Vincken, J.-P.

ID	(Iso)flavonoid subclass	Name	KS split	MRSA MIC (µg/mL)	MRSA MIC (µM)	% Prediction error	Reference
**37**	**Isoflavanone**	**Eryvarin Z**	EV	50	122	9.0	[16]
**38**	**Isoflavanone**	**Eryzerin A**	TR	25	61	5.7	[15]
39	Isoflavanone	Eryzerin B	-	>50	n.a.	n.a	[14,17]
40	Isoflavanone	Glicoisoflavanone	TR	64	167	8.9	[8]
41	Isoflavanone	Glyasperin F	TR	64	181	2.0	[8]
42	Isoflavanone	Glycyrrhisoflavanone	TR	64	174	1.3	[8]
43	Isoflavanone	Licoisoflavanone	TR	32	90	3.1	[8]
**44**	**Isoflavanone**	**Orientanol E**	EV	13	31	6.5	[16]
**45**	**Isoflavanone**	**Orientanol F**	TE	12.5	31	5.0	[13]
**46**	**Isoflavanone**	**Prenyl-costarone**	EV	50	115	10.1	[18]
47	Isoflavene	Bidwillol A	EV	13	38	3.4	[5]
48	Isoflavene	Erypoegin A	TR	25	74	9.8	[20]
49	Isoflavene	Glabrene	TR	25	78	2.6	This study
**50**	**Isoflavone**	**6,8-diprenylgenistein**	TR	9	23	1.3	This study
51	Isoflavone	2,3-dehydrokievitone	-	>100	n.a.	n.a	This study
**52**	**Isoflavone**	**Chandalone**	TR	16	40	4.2	[11]
**53**	**Isoflavone**	**Derrisisoflavone A**	TR	4	10	5.9	[11]
54	Isoflavone	Erysubin B	-	>25	n.a.	n.a	[21]
55	**Isoflavone**	**Erysubin F**	Outlier	100	256	n.a	[6]
56	Isoflavone	Gancaonin G	TE	16	45	2.6	[8]
57	Isoflavone	Glicoricone	TR	64	174	6.3	[8]
58	Isoflavone	Glisoflavone	TR	64	174	0.3	[8]
59	Isoflavone	Glycyrrhisoflavone	TR	64	181	6.1	[8]
**60**	**Isoflavone**	**Isoangustone A**	TE	16	38	3	[8]
61	Isoflavone	Isochandalone	-	>256	n.a.	n.a	[11]
**62**	**Isoflavone**	**Isolupalbigenin**	TR	3	8	6	[21]
63	Isoflavone	Iso-osajin	-	>100	n.a.	n.a	This study
64	Isoflavone	Isowighteone	TE	32	95	5.1	[8]
65	Isoflavone	Licoisoflavone A	TE	25	71	5.0	This study
66	Isoflavone	Licoisoflavone B	TR	13	35	7.1	[9]
**67**	**Isoflavone**	**Lupalbigenin**	TR	4	10	5.5	[11]
68	Isoflavone	Lupiwighteone	-	>100	n.a.	n.a	This study
69	Isoflavone	Luteone	TR	25	71	2.1	This study
70	Isoflavone	Neobavaisoflavone	TR	37.5	116	8.1	This study
71	Isoflavone	Scandinone	-	>256	n.a.	n.a	[11]
72	Isoflavone	Semilicoisoflavone B	TR	64	182	4.5	[8]
73	Isoflavone	Strobilliferylin	*(cr.2)	36	76	18	[22]
74	Isoflavone	Wighteone (Erythrinin B)	TR	15.6	46	2.6	This study
**75**	**Pterocarpan**	**2-(dimethylallyl)-6a-hydroxyphaseollidin**	TR	12.5	31	5.2	[13]
76	Pterocarpan	Anhydrotuberosin	-	>100	n.a.	n.a	This study
77	Pterocarpan	Cristacarpin	TR	100	282	4.9	[6]
**78**	**Pterocarpan**	**Erybraedin A/ 4-prenylphaseollidin**	TR	3	8	5.8	[14,17]
**79**	**Pterocarpan**	**Erystagallin A**	TE	25	59	3	[13]
80	Pterocarpan	Erythribyssin A	TR	32	87	6.8	[23]
**81**	**Pterocarpan**	**Eryzerin E**	TR	25	59	2.8	[15]
82	Pterocarpan	Fuscacarpan A	-	>200	n.a.	n.a	[23]
83	Pterocarpan	Fuscacarpan B	-	>200	n.a.	n.a	[23]
84	Pterocarpan	Fuscacarpan C	-	>200	n.a.	n.a	[23]
85	Pterocarpan	Glyceofuran	-	>100	n.a.	n.a	This study
86	Pterocarpan	Glyceollidin II	TE	44	129	0.0	This study
87	Pterocarpan	Glyceollin I	Outlier	100	296	n.a	This study
88	Pterocarpan	Glyceollin II	TR	150	443	11.4	This study
89	Pterocarpan	Glyceollin III	Outlier	100	296	n.a	This study
90	Pterocarpan	Glyceollin IV	TR	43.75	123	3.8	This study
91	Pterocarpan	Glyceollin V	-	>100	n.a.	n.a	This study
92	Pterocarpan	Glycyrol	-	>128	n.a.	n.a	[8]

ID	(Iso)flavonoid subclass	Name	KS split	MRSA MIC (µg/mL)	MRSA MIC (µM)	% Prediction error	Reference
93	Pterocarpan	Orientanol B	TR	6	18	10.1	[13]
**94**	**Pterocarpan**	**Orientanol C**	TR	25	64	9	[13]
95	Pterocarpan	Phaseollidin	TR	50	154	9.3	[13]
96	Pterocarpan	Phaseollin	TR	25	78	1.7	[13]
97	Pterocarpan	Sandwicensin	TE	16	47	3.4	[23]
98	Pterocarpene	Dehydroglyceollidin II	TE	22	68	4.1	This study
99	Pterocarpene	Dehydroglyceollin I	TE	16	49	2.6	This study
100	Pterocarpene	Dehydroglyceollin II	TR	19	59	1.8	This study
101	Pterocarpene	Dehydroglyceollin III	-	>100	n.a.	n.a	This study
102	Pterocarpene	Dehydroglyceollin IV	TE	44	130	13.8	This study
103	Pterocarpene	Erypoegin H	TR	13	39	1.8	[6]
**104**	**Pterocarpene**	**Erycristagallin**	TE	6	16	2.0	[13]
105	Pterocarpene	Eryvarin D	TR	25	74	4.8	[6]
**106**	**Pterocarpene**	**Eryvarin W**	TR	3	8	3.0	[19]

Supplementary files 'Insights into the molecular properties underlying antibacterial activity of prenylated (iso)flavonoids against MRSA' by Kalli, S., Araya-Cloutier, C., Hageman, J. and Vincken, J.-P.

Table S2. List of descriptors

No.	Descriptors	Definition
1	*a_acc*	Number of hydrogen bond acceptor atoms (not counting acidic atoms but counting atoms that are both hydrogen bond donors and acceptors such as -OH).
2	*a_aro*	Number of aromatic atoms.
3	*a_donacc*	Number of hydrogen bond donor plus number of hydrogen bond acceptor atoms.
4	*a_ICM*	Atom information content (mean). This is the entropy of the element distribution in the molecule (including implicit hydrogens but not lone pair pseudo-atoms). Let n_i be the number of occurrences of atomic number I in the molecule. Let $p_i = n_i / n$ where n is the sum of the n_i. The value of *a_ICM* is the negative of the sum over all I of $p_i \log p_i$.
5	*ASA-*	Water accessible surface area of all atoms with negative partial charge (strictly less than 0).
6	*ASA_H*	Water accessible surface area of all hydrophobic ($\|q_i\|<0.2$) atoms.
7	*ASA_P*	Water accessible surface area of all polar ($\|q_i\|>=0.2$) atoms.
8	*ASA+*	Water accessible surface area of all atoms with positive partial charge (strictly greater than 0).
9	*b_double*	Number of double bonds. Aromatic bonds are not considered to be double bonds.
10	*b_heavy*	Number of bonds between heavy atoms.
11	*b_rotN*	Number of rotatable bonds. A bond is rotatable if it has order 1, is not in a ring, and has at least two heavy neighbours.
12	*balabanJ*	Balaban's connectivity topological index.
13	*CASA-*	Negative charge weighted surface area, ASA- times max ($q_i < 0$).
14	*CASA+*	Positive charge weighted surface area, ASA+ times max ($q_i < 0$).
15	*chiral*	Number of chiral centres.
16	*DASA*	Absolute value of the difference between ASA+ and ASA-.
17	*dens*	Mass density: molecular weight divided by van der Waals volume as calculated in the *vol* descriptor.
18	*diameter*	Largest value in the distance matrix.
19	*dipole*	Dipole moment calculated from the partial charges of the molecule.
20	*E_ang*	Angle bend potential energy. In the Potential Setup panel, the term enable (Bonded) flag is ignored, but the term weight is applied.
21	*E_ele*	Electrostatic component of the potential energy. In the Potential Setup panel, the term enable flag is ignored, but the term weight is applied.
22	*E_oop*	Out-of-plane potential energy. In the Potential Setup panel, the term enable (Bonded) flag is ignored, but the term weight is applied.
23	*E_sol*	Solvation energy. In the Potential Setup panel, the term enable parameter (Solvation menu) is ignored, but the term weight is applied.
24	*E_str*	Bond stretch potential energy. In the Potential Setup panel, the term enable (Bonded) flag is ignored, but the term weight is applied.
25	*E_strain*	Local strain energy: the current energy minus the value of the energy at a near local minimum. The current energy is calculated as for the E descriptor. The local minimum energy is the value of the E descriptor after first performing an energy minimization. Current chirality is preserved and charges are left undisturbed during minimization. The structure in the dataset is not modified (results of the minimization are discarded).
26	*E_tor*	Torsion (proper and improper) potential energy. In the Potential Setup panel, the term enable (Bonded) flag is ignored, but the term weight is applied.
27	*E_vdw*	van der Waals component of the potential energy. In the Potential Setup panel, the term enable flag is ignored, but the term weight is applied.
28	*FASA-*	Fractional ASA- calculated as ASA- / ASA.
29	*FASA_H*	Fractional ASA_H calculated as ASA_H / ASA.
30	*FASA+*	Fractional ASA+ calculated as ASA+ / ASA.
31	*FCASA-*	Fractional CASA- calculated as CASA- / ASA.
32	*FCASA+*	Fractional CASA+ calculated as CASA+ / ASA.

33	*glob*	Globularity, or inverse condition number smallest eigenvalue divided by the largest eigenvalue) of the covariance matrix of atomic coordinates. A value of 1 indicates a perfect sphere while a value of 0 indicates a two- or one-dimensional object.
34	*h_ema*	Sum of hydrogen bond acceptor strengths
35	*h_emd*	Sum of hydrogen bond donor strengths.
36	*h_emd_C*	Sum of hydrogen bond donor strengths of carbon atoms.
37	*h_logD*	The octanol/water distribution coefficient at pH 7 calculated as a state average: log sum ($10^{h\_logPi\ -\ pCi}$).
38	*h_pavgQ*	The average total charge sum ($Q_i\ 10^{-pCi}$) where Q_i is the total formal charge of state *i*.
39	*h_pKa*	The pKa of the reaction that removes a proton from the ensemble of states with a hydrogen count equal to the input structure;14 is reported if there are no states with fewer hydrogens than the input.
40	*h_pstrain*	The strain energy (kcal/mol) needed to convert all protonation states into the input protonation state: (kT ln 10) (pC1 + log sum (10-pCi))
41	*KierFlex*	Kier molecular flexibility index: (KierA1) (KierA2) / *n*
42	*lip_acc*	The number of O and N atoms.
43	*lip_don*	The number of OH and NH atoms
44	*logP(o/w)*	Log of the octanol/water partition coefficient (including implicit hydrogens). This property is calculated from a linear atom type model [LOGP 1998] with r^2 = 0.931, RMSE=0.393 on 1,827 molecules.
45	*logS*	Log of the aqueous solubility (mol/L). This property is calculated from an atom contribution linear atom type model [Hou 2004] with r^2 = 0.90, ~1,200 molecules.
46	*npr1*	Normalized PMI ratio pmi1/pmi3.
47	*npr2*	Normalized PMI ratio pmi2/pmi3.
48	*PC-*	Total negative partial charge: the sum of the negative qi
49	*PEOE_PC-*	Total negative partial charge: the sum of the negative q_i. Q_PC- is identical to PC- which has been retained for compatibility.
50	*PEOE_RPC-*	Relative negative partial charge: the smallest negative qi divided by the sum of the negative qi. Q_RPC- is identical to RPC- which has been retained for compatibility.
51	*PEOE_RPC+*	Relative positive partial charge: the largest positive qi divided by the sum of the positive qi. Q_RPC+ is identical to RPC+ which has been retained for compatibility.
52	*PEOE_VSA_FHYD*	Fractional hydrophobic van der Waals surface area. This is the sum of the vi such that |qi| is less than or equal to 0.2 divided by the total surface area. The vi are calculated using a connection table approximation.
53	*PEOE_VSA_FNEG*	Fractional negative van der Waals surface area. This is the sum of the vi such that qi is negative divided by the total surface area. The vi are calculated using a connection table approximation.
54	*PEOE_VSA_HYD*	Total hydrophobic van der Waals surface area. This is the sum of the vi such that |qi| is less than or equal to 0.2. The vi are calculated using a connection table approximation.
55	*PEOE_VSA_NEG*	Total negative van der Waals surface area. This is the sum of the vi such that qi is negative. The vi are calculated using a connection table approximation.
56	*PEOE_VSA_POS*	Total positive van der Waals surface area. This is the sum of the vi such that qi is non-negative. The vi are calculated using a connection table approximation.
57	*PEOE_VSA_PPOS*	Total positive polar van der Waals surface area. This is the sum of the vi such that qi is greater than 0.2. The vi are calculated using a connection table approximation.
58	*PEOE_VSA+0*	Sum of vi where qi is in the range [0.00,0.05).
59	*PEOE_VSA+1*	Sum of vi where qi is in the range [0.05,0.10).
60	*PEOE_VSA+2*	Sum of vi where qi is in the range [0.10,0.15).
61	*PEOE_VSA-0*	Sum of vi where qi is in the range [-0.05,0.00).
62	*PEOE_VSA-1*	Sum of vi where qi is in the range [-0.10,-0.05).
63	*PM3_dipole*	The dipole moment calculated using the PM3 Hamiltonian.
64	*PM3_E*	The total energy (kcal/mol) calculated using the PM3 Hamiltonian [MOPAC].
65	*PM3_HF*	The heat of formation (kcal/mol) calculated using the PM3 Hamiltonian [MOPAC].
66	*PM3_IP*	The ionization potential (kcal/mol) calculated using the PM3 Hamiltonian [MOPAC].

67	*PM3_LUMO*	The energy (eV) of the Lowest Unoccupied Molecular Orbital calculated using the PM3 Hamiltonian [MOPAC].
68	*pmi1*	First diagonal element of diagonalized moment of inertia tensor.
69	*pmi3*	Third diagonal element of diagonalized moment of inertia tensor.
70	*radius*	If r_i is the largest matrix entry in row *i* of the distance matrix *D*, then the radius is defined as the smallest of the r_i [Petitjean 1992].
71	*rgyr*	Radius of gyration.
72	*rings*	The number of rings.
73	*RPC-*	Relative negative partial charge: the smallest negative qi divided by the sum of the negative qi. Q_RPC- is identical to RPC- which has been retained for compatibility.
74	*RPC+*	Relative positive partial charge: the largest positive qi divided by the sum of the positive qi. Q_RPC+ is identical to RPC+ which has been retained for compatibility.
75	*SlogP*	Log of the octanol/water partition coefficient (including implicit hydrogens). This property is an atomic contribution model [Crippen 1999] that calculates logP from the given structure; i.e., the correct protonation state (washed structures). Results may vary from the logP(o/w) descriptor. The training set for SlogP was ~7000 structures.
76	std_dim1	Standard dimension 1: the square root of the largest eigenvalue of the covariance matrix of the atomic coordinates. A standard dimension is equivalent to the standard deviation along a principal component axis.
77	std_dim2	Standard dimension 2: the square root of the second largest eigenvalue of the covariance matrix of the atomic coordinates. A standard dimension is equivalent to the standard deviation along a principal component axis.
78	std_dim3	Standard dimension 3: the square root of the third largest eigenvalue of the covariance matrix of the atomic coordinates. A standard dimension is equivalent to the standard deviation along a principal component axis.
79	*vol*	van der Waals volume calculated using a grid approximation (spacing 0.75 A).
80	*vsa_acc*	Approximation to the sum of VDW surface areas (Å2) of pure hydrogen bond acceptors (not counting atoms that are both hydrogen bond donors and acceptors such as -OH).
81	*vsa_pol*	Approximation to the sum of VDW surface areas (Å2) of polar atoms (atoms that are both hydrogen bond donors and acceptors), such as -OH.
82	*vsurf_A*	Amphiphilic moment
83	*vsurf_CP*	Critical packing parameter
84	*vsurf_CW2*	Capacity factor at −0.5 kcal/mol.
85	*vsurf_CW3*	Capacity factor at −1.0 kcal/mol.
86	*vsurf_D4*	Hydrophobic volume at -0.8 kcal/mol.
87	*vsurf_D6*	Hydrophobic volume at -1.2 kcal/mol.
88	*vsurf_D7*	Hydrophobic volume at -1.4 kcal/mol.
89	*vsurf_D8*	Hydrophobic volume at -1.6 kcal/mol.
90	*vsurf_DD12*	Contact distances. vsurf_EDmin1, vsurf_EDmin2 distance.
91	*vsurf_DD13*	vsurf_EDmin1, vsurf_EDmin3 distance.
92	*vsurf_DD23*	vsurf_EDmin2, vsurf_EDmin3 distance.
93	*vsurf_DW12*	vsurf_EWmin1, vsurf_EWmin2 distance.
94	*vsurf_DW13*	vsurf_EWmin1, vsurf_EWmin3 distance.
95	*vsurf_DW23*	vsurf_EWmin2, vsurf_EWmin3 distance
96	*vsurf_EDmin1*	Lowest hydrophobic energy.
97	*vsurf_EWmin1*	Lowest hydrophilic energy.
98	*vsurf_G*	Surface globularity
99	*vsurf_HB1*	H-bond donor capacity at -0.2 kcal/mol.
100	*vsurf_HB5*	H-bond donor capacity at -0.5 kcal/mol.
101	*vsurf_HL1*	First hydrophilic-lipophilic balance.
102	*vsurf_ID1*	Hydrophobic interaction energy (integy) moment at -0.2 kcal/mol.
103	*vsurf_ID4*	Hydrophobic integy moment at -0.8 kcal/mol.

104	*vsurf_ID6*	Hydrophobic integy moment at -1.2 kcal/mol.
105	*vsurf_ID7*	Hydrophobic integy moment at -1.4 kcal/mol.
106	*vsurf_ID8*	Hydrophobic integy moment at -1.6 kcal/mol.
107	*vsurf_IW1*	Hydrophilic integy moment at -0.2 kcal/mol.
108	*vsurf_IW2*	Hydrophilic integy moment at -0.5 kcal/mol.
109	*vsurf_IW3*	Hydrophilic integy moment at -1.0 kcal/mol.
110	*vsurf_IW6*	Hydrophilic integy moment at −4.0 kcal/mol
111	*vsurf_IW7*	Hydrophilic integy moment at -5.0 kcal/mol.
112	*vsurf_R*	Surface rugosity
113	*vsurf_W1*	Hydrophilic volume at −0.2 kcal mol-1
114	*vsurf_W3*	Hydrophilic volume at -1.0 kcal/mol.
115	*vsurf_W7*	Hydrophilic volume at -5.0 kcal/mol.
116	*vsurf_Wp1*	Polar volume
117	*vsurf_Wp2*	Polar volume at -0.5 kcal/mol.
118	*vsurf_Wp3*	Polar volume at -1.0 kcal/mol.
119	*vsurf_Wp4*	Polar volume at -2.0 kcal/mol.
120	*vsurf_Wp6*	Polar volume at -4.0 kcal/mol.
121	*vsurf_Wp7*	Polar volume at -5.0 kcal/mol.

Supplementary files 'Insights into the molecular properties underlying antibacterial activity of prenylated (iso)flavonoids against MRSA' by Kalli, S., Araya-Cloutier, C., Hageman, J. and Vincken, J.-P.

Table S3. Prenylated (iso)flavonoids with their respective reported and predicted activity (MIC, µg/mL) against Gram-positive bacteria, other than MRSA. The structures of compounds **G1-G39** can be found in **Figure S5**. Molecules of which the level of activity (high, moderate, low) was not predicted correctly by the corresponding model are highlighted in red. Di-prenylated (iso)flavonoids are indicated in **bold**. Molecules in grey have been also tested against MRSA (**Table S1**). Molecules are sorted based on subclass and ordered alphabetically within each subclass.

ID	(Iso)flavonoid subclass	Name	Highest reported MIC	Predicted MIC	Gram+ bacterium	Reference
5	2-arylbenzofuran	Moracin C	12.5	12	*B. subtilis/ S. aureus/* MRSA	[9]
7	3-arylcoumarin	Glycycoumarin	16	36	*S. aureus/* MRSA	[8]
8	3-arylcoumarin	Glycyrin	128	40	*B. subtilis/ S. aureus/ MRSA*	[8,10]
G1	Flavanone	(2S)-5,7-dihydroxy-4'-methoxy-8-prenylflavanone	32	47	*B. subtilis*	[24]
**G2**	**Flavanone**	**2(S)-5′-(1′′′,1′′′-Dimethylallyl)-8-(3′′,3′′-dimethylallyl)- 2′,4′,5,7-tetrahydroxyflavanone**	8	28	*S. aureus*	[25]
G3	Flavanone	5-Dehydroxyparatocarpin K	12	35	*S. epidermis*	[26]
G4	Flavanone	5-Methylsophoraflavanone B (isoxanthohumol)	20	40	*S. epidermis*	[27]
G5	Flavanone	Abyssinone I	25	32	*B. subtilis/S. aureus*	[28]
G6	Flavanone	Abyssinone II	50	23	*B. subtilis/S. aureus*	[28]
**G7**	**Flavanone**	**Abyssinone III**	100	11	*B. subtilis/S. aureus*	[28]
**G8**	**Flavanone**	**Abyssinone IV**	25	10	*B. subtilis/S. aureus*	[28]
**G9**	**Flavanone**	**Abyssinone V**	50	15	*B. subtilis/S. aureus*	[28]
G10	Flavanone	Bavachin (coryfolin)	12	23	*S. epidermis*	[26]
G11	Flavanone	Bavachinin	6	20	*S. epidermis*	[26]
**G12**	**Flavanone**	**Burttinone**	62	107	*S. aureus*	[29]
G13	Flavanone	Citflavanone	>100	59	*E. facealis*	[30]
**G14**	**Flavanone**	**Erythrisenegalone**	50	23	*E. facealis*	[30]
G15	Flavanone	Kushenol S	5	37	*B. subtilis*	[31]
G16	Flavanone	Kushenol V	10	30	*B. subtilis*	[31]
G17	Flavanone	Leachianone G	2	27	*B. subtilis*	[31]
**G18**	**Flavanone**	**Lonchocarpol A**	6	22	*E. facealis*	[30]
**G19**	**Flavanone**	**Lupinifolin**	6	30	*B. subtilis/ E.facealis*	[30]
G20	Flavanone	Macatrichocarpin A	10	23	*B. subtilis*	[32]
G21	Flavanone	Macatrichocarpin B	18	21	*B. subtilis*	[32]
20	Isoflavan	Eryzerin C	10	20	*B. subtilis/ S. aureus / S. epidermis/* MRSA	[33]
22	Isoflavan	Glabridin	31	20	*S. aureus/ S. mutans/ B. subtilis/ E. facealis/* MRSA	[8,10,34]
24	Isoflavan	Glyasperin D	16	21	*B. subtilis / S. aureus/* MRSA	[8,10]
**27**	**Isoflavan**	**Licoricidin**	16	14	*B. subtilis/ S. aureus/* MRSA	[8,10]
**G22**	**Isoflavan**	**Licorisoflavan A (5-O-Methyllicoricidin)**	4	15	*S. mutans*	[24]
**31**	**Isoflavanone**	**3'-dimethylallyl-kievitone**	8	11	*S. aureus/* MRSA	[8]
**G23**	**Isoflavanone**	**Dalversinol A**	31	13	*S. aureus*	[25]
40	Isoflavanone	Glicoisoflavanone	32	30	*S. aureus/* MRSA	[8]
41	Isoflavanone	Glyasperin F	32	76	*S. aureus/* MRSA	[8]
42	Isoflavanone	Glycyrrhisoflavanone	32	57	*S. aureus/* MRSA	[8]
43	Isoflavanone	Licoisoflavanone	32	43	*S. aureus/* MRSA	[8]

Supplementary files 'Insights into the molecular properties underlying antibacterial activity of prenylated (iso)flavonoids against MRSA' by Kalli, S., Araya-Cloutier, C., Hageman, J. and Vincken, J.-P.

ID	(Iso)flavonoid subclass	Name	Highest reported MIC	Predicted MIC	Gram+ bacterium	Reference
G24	Isoflavanone	Lysisteisoflavone	62	39	*S. aureus*	[33]
G25	Isoflavanone	Sophoraisoflavanone A	20	57	*S. epidermis*	[27]
49	Isoflavene	Glabrene	12.5	20	*B. subtilis/ MRSA*	[10]
**50**	**Isoflavone**	**6,8-Diprenyl genistein**	2	8	*S. mutans/ S. aureus/* MRSA	[8,35]
G26	Isoflavone	Alpinumisoflavone	31	34	*S. aureus*	[33]
**G27**	**Isoflavone**	**Auriculasin**	2	15	*B. subtilis*	[36]
G28	**Isoflavone**	**Auriculatin**	2	11	*B. subtilis*	[36]
**52**	**Isoflavone**	**Chandalone**	128	10	*S. aureus/* MRSA	[11]
**53**	**Isoflavone**	**Derrisisoflavone**	16	8	*S. aureus/* MRSA	[11]
G29	Isoflavone	Erythrinin A	6	24	*S. epidermis*	[26]
56	Isoflavone	Gancaonin G	125	21	*S. mutans/ S. aureus*	[8,35]
57	Isoflavone	Glicoricone	64	37	*S. aureus/* MRSA	[8]
58	Isoflavone	Glisoflavone	32	66	*S. aureus/* MRSA	[8]
59	Isoflavone	Glycyrrhisoflavone	32	38	*S. aureus/* MRSA	[8]
**60**	**Isoflavone**	**Isoangustone A**	16	12	*S. aureus/* MRSA	[8]
64	Isoflavone	Isowighteone	16	20	*S. aureus/* MRSA	[8]
66	Isoflavone	Licoisoflavone B	12.5	26	*S. aureus/* MRSA	[10]
**67**	**Isoflavone**	**Lupalbigenin**	4	8	*S. aureus/* MRSA	[11]
**G30**	**Isoflavone**	**Millexatin F**	2	11	*B. subtilis*	[36]
**G31**	**Isoflavone**	**Scandenone**	8	11	*B. subtilis*	[37]
72	Isoflavone	Semilicoisoflavone B	32	44	*S. aureus*	[8]
**G32**	**Pterocarpan**	**1-Methoxyficifolinol**	4	14	*S. mutans*	[24]
77	Pterocarpan	Cristacarpin	412	67	*B. subtilis/ S. aureus / S. epidermis/* MRSA	[23,33]
**78**	**Pterocarpan**	**Erybraedin A/ 4-prenylphaseollidin**	2	6	*B. subtilis/ S. aureus / S. epidermis/* MRSA	[33]
**G33**	**Pterocarpan**	**Erybraedin B**	12	8	*S. aureus*	[38]
**G34**	**Pterocarpan**	**Erybraedin C**	12	6	*S. aureus*	[38]
**G35**	**Pterocarpan**	**Erycristin**	6	8	*S. aureus*	[38]
80	Pterocarpan	Erythribyssin A	64	60	*S. aureus/ MRSA*	[23]
**G36**	**Pterocarpan**	**Erythrabyssin II**	3	7	*B. subtilis*	[28]
G37	Pterocarpan	Isoneorutaneol	25	30	*S. aureus*	[38]
95	Pterocarpan	Phaseollidin	50	22	*S. aureus/ B. subtilis/* MRSA	[28]
96	Pterocarpan	Phaseollin	12.5	29	*S. aureus/ B. subtilis/* MRSA	[28]
97	Pterocarpan	Sandwicensin	100	22	*S. aureus/ B. subtilis / E. facealis/* MRSA	[23,30]
105	Pterocarpene	Eryvarin D	4	16	*S. aureus/* MRSA	[23]
**G38**	**Pterocarpene**	**Glycyrrhizol A**	1	7	*S. mutans*	[35]
G39	Pterocarpene	Glycyrrhizol B	32	17	*S. mutans*	[35]

Supplementary files 'Insights into the molecular properties underlying antibacterial activity of prenylated (iso)flavonoids against MRSA' by Kalli, S., Araya-Cloutier, C., Hageman, J. and Vincken, J.-P.

Table S4. Most frequent descriptors used in the top QSAR models and their significance (n: 2-7).

**Descriptor**	***p-value***
h_emd_C	$2.2e^{-04}$-$1.7e^{-02}$
vsurf_D4	1.0-$1.9e^{-10}$
PEOE_VSA_PPOS	$3.6e^{-06}$ – $1.4e^{-05}$
vsurf_CW3	$4.7e^{-13}$-$4.1e^{-09}$
vsurf_IW7	$3.5e^{-04}$-$3.9e^{-01}$
E_vdw	$5.8e^{-03}$
h_pavgQ	$1.7e^{-06}$-$1.2e^{-04}$
PEOE_VSA+2	$4.2e^{-03}$ -$1.6e^{-01}$
vsurf_DD12	$4.7e^{-02}$-$1.5e^{-01}$
PM3_IP	$7.1e^{-02}$

Table S5. Prenylated (iso)flavonoids mapping all features of the pharmacophore model of MRSA. [a] RMSD is the root-mean-square deviation which determines the quality of fitting of each prenylated (iso)flavonoid to the pharmacophore features. [b] True (+) and false (-) positive. Di-prenylated (iso)flavonoids are indicated **in bold**.

ID	(Iso)flavonoid subclass	Name	MIC (μg/mL)	RMSD[a]	Outcome[b]
3	2-arylbenzofuran	Eryvarin U	12	0.64	-
4	2-arylbenzofuran	Licocoumarone	16	0.46	+
5	2-arylbenzofuran	Moracin C	12	0.5	+
7	3-arylcoumarin	Glycycoumarin	16	0.76	+
8	3-arylcoumarin	Glycyrin	128	0.62	+
16	Isoflavan	3'-OH-4'-O-methylglabridin	16	0.68	+
18	Isoflavan	Eryvarin C	25	0.4	+
21	Isoflavan	Eryzerin D	12	0.4	+
22	Isoflavan	Glabridin	16	0.63	+
23	Isoflavan	Glyasperin C	16	0.63	+
24	Isoflavan	Glyasperin D	16	0.78	+
**25**	**Isoflavan**	**Hispaglabridin A**	50	0.64	+
**27**	**Isoflavan**	**Licoricidin**	16	0.64	-
28	Isoflavan	Phaseollin isoflavan	50	0.41	-
**34**	**Isoflavanone**	**Diprenyl-costarone**	6	0.66	+
**35**	**Isoflavanone**	**Eryvarin V**	25	0.86	+
**46**	**Isoflavanone**	**Prenyl-costarone**	50	0.68	-
49	Isoflavene	Glabrene	25	0.39	+
**50**	**Isoflavone**	**6,8-diprenylgenistein**	9	0.66	+
**52**	**Isoflavone**	**Chandalone**	16	0.56	+
**53**	**Isoflavone**	**Derrisisoflavone A**	4	0.68	+
56	Isoflavone	Gancaonin G	16	0.73	+
**60**	**Isoflavone**	**Isoangustone A**	16	0.7	+
**62**	**Isoflavone**	**Isolupalbigenin**	3	0.69	-
65	Isoflavone	Licoisoflavone A	25	0.59	+
66	Isoflavone	Licoisoflavone B	12	0.21	+
**67**	**Isoflavone**	**Lupalbigenin**	4	0.66	-
69	Isoflavone	Luteone	25	0.65	+
72	Isoflavone	Semilicoisoflavone B	64	0.3	-
74	Isoflavone	Wighteone/Erythrinin B	16	0.68	+
**75**	**Pterocarpan**	**2-(dimethylallyl)-6a-hydroxyphaseollidin**	12	0.39	+
77	Pterocarpan	Cristacarpin/Erythrabissin I	100	0.59	+
**79**	**Pterocarpan**	**Erystagallin A**	25	0.59	+
**81**	**Pterocarpan**	**Eryzerin E**	25	0.75	+
96	Pterocarpan	Phaseollin	25	0.92	+
97	Pterocarpan	Sandwicensin	16	0.6	+
99	Pterocarpene	Dehydroglyceollin I	16	0.63	-
105	Pterocarpene	Eryvarin D	25	0.6	+

Supplementary files 'Insights into the molecular properties underlying antibacterial activity of prenylated (iso)flavonoids against MRSA' by Kalli, S., Araya-Cloutier, C., Hageman, J. and Vincken, J.-P.

Table S6. Prenylated (iso)flavonoids mapping all features of the pharmacophore model of L. monocytogenes [39]. [a] RMSD is the root-mean-square deviation which determines the quality of fitting of each prenylated (iso)flavonoid to the pharmacophore features. [b] True (+) and false (-) positive. Di-prenylated (iso)flavonoids are indicated **in bold**.

ID	(Iso)flavonoid subclass	Name	MIC (μg/mL)	RMSD[a]	Outcome[b]
3	2-arylbenzofuran	Eryvarin U	12	0.72	+
5	2-arylbenzofuran	Moracin C	12	0.73	+
7	3-arylcoumarin	Glycycoumarin	16	0.65	+
8	3-arylcoumarin	Glycyrin	128	0.68	-
12	Flavanone	6-prenyl-naringenin	38	0.73	-
16	Isoflavan	3'-OH-4'-O-methylglabridin	16	0.52	+
17	Isoflavan	4'-O-methylglabridin	10	0.52	+
20	Isoflavan	Eryzerin C	6	0.58	+
22	Isoflavan	Glabridin	16	0.52	+
23	Isoflavan	Glyasperin C	16	0.7	+
24	Isoflavan	Glyasperin D	16	0.48	+
**25**	**Isoflavan**	**Hispaglabridin A**	50	0.51	-
**27**	**Isoflavan**	**Licoricidin**	16	0.7	+
29	Isoflavan	Phaseollin isoflavan	50	0.53	-
**34**	**Isoflavanone**	**Diprenyl-costarone**	6	0.73	+
40	Isoflavanone	Glicoisoflavanone	64	0.47	-
**45**	**Isoflavanone**	**Orientanol F**	12	0.75	+
48	Isoflavene	Erypoegin A	25	0.56	+
**50**	**Isoflavone**	**6,8-diprenylgenistein**	9	0.25	+
**52**	**Isoflavone**	**Chandalone**	16	0.36	+
**53**	**Isoflavone**	**Derrisisoflavone A**	4	0.55	+
56	Isoflavone	Gancaonin G	16	0.42	+
57	Isoflavone	Glicoricone	64	0.66	-
58	Isoflavone	Glisoflavone	64	0.82	-
**60**	**Isoflavone**	**Isoangustone A**	16	0.26	+
65	Isoflavone	Licoisoflavone A	25	0.47	+
66	Isoflavone	Licoisoflavone B	12	0.45	+
**67**	**Isoflavone**	**Lupalbigenin**	4	0.25	+
69	Isoflavone	Luteone	25	0.37	+
74	Isoflavone	Wighteone/Erythrinin B	16	0.33	+
**78**	**Pterocarpan**	**Erybraedin A/ 4-prenylphaseollidin**	3	0.52	+
**81**	**Pterocarpan**	**Eryzerin E**	25	0.51	+
90	Pterocarpan	Glyceollin IV	44	0.52	-
93	Pterocarpan	Orientanol B	6	0.66	+
102	Pterocarpene	Dehydroglyceollin IV	44	0.49	-

Supplementary files 'Insights into the molecular properties underlying antibacterial activity of prenylated (iso)flavonoids against MRSA' by Kalli, S., Araya-Cloutier, C., Hageman, J. and Vincken, J.-P.

References

[1] Gramatica, P. Principles of QSAR models validation: internal and external. *QSAR & combinatorial science* **26**, 694-701 (2007).

[2] Keepers, T. R., Gomez, M., Biek, D., Critchley, I. & Krause, K. M. Effect of *in vitro* testing parameters on ceftazidime-avibactam minimum inhibitory concentrations. *International Scholarly Research Notices* **2015** (2015).

[3] Brennan-Krohn, T., Smith, K. P. & Kirby, J. E. The poisoned well: enhancing the predictive value of antimicrobial susceptibility testing in the era of multidrug resistance. *Journal of Clinical Microbiology* **55**, 2304-2308 (2017).

[4] Li, J., Beuerman, R. W. & Verma, C. S. Molecular insights into the membrane affinities of model hydrophobes. *ACS Omega* **3**, 2498-2507 (2018).

[5] Tanaka, H., Sudo, M., Kawamura, T., Sato, M., Yamaguchi, R., Fukai, T., Sakai, E. & Tanaka, N. Antibacterial constituents from the roots of *Erythrina herbacea* against methicillin-resistant *Staphylococcus aureus*. *Planta Medica* **76**, 916-919 (2010).

[6] Tanaka, H., Sato, M., Oh-Uchi, T., Yamaguchi, R., Etoh, H., Shimizu, H., Sako, M. & Takeuchi, H. Antibacterial properties of a new isoflavonoid from *Erythrina poeppigiana* against methicillin-resistant *Staphylococcus aureus*. *Phytomedicine* **11**, 331-337 (2004).

[7] Tanaka, H., Sudo, M. & Hirata, M. Two new isoflavonoids and a new 2-arylbenzofuran from the roots of *Erythrina variegata*. *Heterocycles* **65**, 871-877 (2005).

[8] Hatano, T., Shintani, Y., Aga, Y., Shiota, S., Tsuchiya, T. & Yoshida, T. Phenolic constituents of licorice. VIII. Structures of glicophenone and glicoisoflavanone, and effects of licorice phenolics on methicillin-resistant *Staphylococcus aureus*. *Chemical & Pharmaceutical Bulletin* **48**, 1286-1292 (2000).

[9] Fukai, T., Kaitou, K. & Terada, S. Antimicrobial activity of 2-arylbenzofurans from *Morus* species against methicillin-resistant *Staphylococcus aureus*. *Fitoterapia* **76**, 708-711 (2005).

[10] Fukai, T., Marumo, A., Kaitou, K., Kanda, T., Terada, S. & Nomura, T. Antimicrobial activity of licorice flavonoids against methicillin-resistant *Staphylococcus aureus*. *Fitoterapia* **73**, 536-539 (2002).

[11] Mahabusarakam, W., Deachathai, S., Phongpaichit, S., Jansakul, C. & Taylor, W. A benzil and isoflavone derivatives from *Derris scandens* Benth. *Phytochemistry* **65**, 1185-1191 (2004).

[12] Mun, S.-H., Kang, O.-H., Joung, D.-K., Kim, S.-B., Seo, Y.-S., Choi, J.-G., Lee, Y.-S., Cha, S.-W., Ahn, Y.-S. & Han, S.-H. Combination therapy of sophoraflavanone B against MRSA: in vitro synergy testing. *Evidence-Based Complementary and Alternative Medicine* **2013** (2013).

[13] Tanaka, H., Sato, M., Fujiwara, S., Hirata, M., Etoh, H. & Takeuchi, H. Antibacterial activity of isoflavonoids isolated from *Erythrina variegata* against methicillin-resistant *Staphylococcus aureus*. *Letters in Applied Microbiology* **35**, 494-498 (2002).

[14] Sato, M., Tanaka, H., Oh-Uchi, T., Fukai, T., Etoh, H. & Yamaguchi, R. Antibacterial activity of phytochemicals isolated from *Erythrina zeyheri* against vancomycin-resistant *enterococci* and their combinations with vancomycin. *Phytotherapy Research: An International Journal Devoted to Pharmacological and Toxicological Evaluation of Natural Product Derivatives* **18**, 906-910 (2004).

[15] Tanaka, H., Oh-Uchi, T., Etoh, H., Sako, M., Asai, F., Fukai, T., Sato, M., Murata, J. & Tateishi, Y. Isoflavonoids from roots of *Erythrina zeyheri*. *Phytochemistry* **64**, 753-758 (2003).

[16] Tanaka, H., Atsumi, I., Hasegawa, M., Hirata, M., Sakai, T., Sato, M., Yamaguchi, R., Tateishi, Y., Tanaka, T. & Fukai, T. Two new isoflavanones from the roots of *Erythrina variegata*. *Natural Product Communications* **10**, 1934578X1501000330 (2015).

[17] Sato, M., Tanaka, H., Yamaguchi, R., Kato, K. & Etoh, H. Synergistic effects of mupirocin and an isoflavanone isolated from Erythrina variegata on growth and recovery of methicillin-resistant *Staphylococcus aureus*. *International journal of antimicrobial agents* **24**, 241-246 (2004).

[18] Tanaka, H., Hattori, H., Oh-Uchi, T., Sato, M., Sako, M., Tateishi, Y. & Rizwani, G. H. Three new isoflavanones from *Erythrina costaricensis*. *Natural Product Research* **23**, 1089-1094 (2009).

[19] Tanaka, H., Atsumi, I., Shirota, O., Sekita, S., Sakai, E., Sato, M., Murata, J., Murata, H., Darnaedi, D. & Chen, I. S. Three new constituents from the roots of *Erythrina variegata* and their antibacterial activity against methicillin-resistant *Staphylococcus aureus*. *Chemistry & biodiversity* **8**, 476-482 (2011).

[20] Sato, M., Tanaka, H., Yamaguchi, R., Oh-Uchi, T. & Etoh, H. *Erythrina poeppigiana*-derived phytochemical exhibiting antimicrobial activity against *Candida albicans* and methicillin-resistant *Staphylococcus aureus*. *Letters in Applied Microbiology* **37**, 81-85 (2003).

[21] Sato, M., Tanaka, H., Tani, N., Nagayama, M. & Yamaguchi, R. Different antibacterial actions of isoflavones isolated from *Erythrina poeppigiana* against methicillin-resistant *Staphylococcus aureus*. *Letters in Applied Microbiology* **43**, 243-248 (2006).

[22] Madan, S., Singh, G. N., Kohli, K., Ali, M., Kumar, Y., Singh, R. M. & Prakash, O. Isoflavonoids from *Flemingia strobilifera* (L) R. Br. roots. *Acta Poloniae Pharmaceutica* **66**, 297-303 (2009).

[23] Innok, P., Rukachaisirikul, T., Phongpaichit, S. & Suksamrarn, A. Fuscacarpans A–C, new pterocarpans from the stems of *Erythrina fusca*. *Fitoterapia* **81**, 518-523 (2010).

[24] Inui, S., Hosoya, T., Shimamura, Y., Masuda, S., Ogawa, T., Kobayashi, H., Shirafuji, K., Moli, R. T., Kozone, I. & Shin-ya, K. Solophenols B–D and Solomonin: New prenylated polyphenols isolated

from propolis collected from the solomon islands and their antibacterial activity. *Journal of agricultural and food chemistry* **60**, 11765-11770 (2012).

[25] Belofsky, G., Percivill, D., Lewis, K., Tegos, G. P. & Ekart, J. Phenolic metabolites of *Dalea versicolor* that enhance antibiotic activity against model pathogenic bacteria. *Journal of natural products* **67**, 481-484 (2004).

[26] Yin, S., Fan, C.-Q., Wang, Y., Dong, L. & Yue, J.-M. Antibacterial prenylflavone derivatives from *Psoralea corylifolia*, and their structure–activity relationship study. *Bioorganic & Medicinal Chemistry* **12**, 4387-4392 (2004).

[27] Sohn, H. Y., Son, K. H., Kwon, C. S., Kwon, G. S. & Kang, S. S. Antimicrobial and cytotoxic activity of 18 prenylated flavonoids isolated from medicinal plants: *Morus alba* L., *Morus mongolica* Schneider, *Broussnetia papyrifera* (L.) Vent, *Sophora flavescens* Ait and *Echinosophora koreensis* Nakai. *Phytomedicine* **11**, 666-672 (2004).

[28] Taniguchi, M. & Kubo, I. Ethnobotanical drug discovery based on medicine men's trials in the African savanna: screening of east African plants for antimicrobial activity II. *Journal of Natural Products* **56**, 1539-1546 (1993).

[29] Chukwujekwu, J., Van Heerden, F. & Van Staden, J. Antibacterial activity of flavonoids from the stem bark of *Erythrina caffra* thunb. *Phytotherapy Research* **25**, 46-48 (2011).

[30] Khaomek, P., Ruangrungsi, N., Saifah, E., Sriubolmas, N., Ichino, C., Kiyohara, H. & Yamada, H. A new pterocarpan from *Erythrina fusca*. *Heterocycles* **63**, 879-884 (2004).

[31] Kuroyanagi, M., Arakawa, T., Hirayama, Y. & Hayashi, T. Antibacterial and antiandrogen flavonoids from *Sophora flavescens*. *Journal of natural products* **62**, 1595-1599 (1999).

[32] Fareza, M. S., Syah, Y. M., Mujahidin, D., Juliawaty, L. D. & Kurniasih, I. Antibacterial flavanones and dihydrochalcones from *Macaranga trichocarpa*. *Zeitschrift für Naturforschung C* **69**, 375-380 (2014).

[33] Sadgrove, N. J., Oliveira, T. B., Khumalo, G. P., Vuuren, S. F. v. & van Wyk, B.-E. Antimicrobial isoflavones and derivatives from *Erythrina* (Fabaceae): structure activity perspective (sar & qsar) on experimental and mined values against *Staphylococcus Aureus*. *Antibiotics* **9**, 223 (2020).

[34] Gupta, V. K., Fatima, A., Faridi, U., Negi, A. S., Shanker, K., Kumar, J., Rahuja, N., Luqman, S., Sisodia, B. S. & Saikia, D. Antimicrobial potential of *Glycyrrhiza glabra* roots. *Journal of ethnopharmacology* **116**, 377-380 (2008).

[35] He, J., Chen, L., Heber, D., Shi, W. & Lu, Q.-Y. Antibacterial Compounds from *Glycyrrhiza uralensis*. *Journal of natural products* **69**, 121-124 (2006).

[36] Raksat, A., Maneerat, W., Andersen, R. J., Pyne, S. G. & Laphookhieo, S. Antibacterial prenylated isoflavonoids from the stems of *Millettia extensa*. *Journal of Natural Products* **81**, 1835-1840 (2018).

[37] Özçelik, B., Orhan, I. & Toker, G. Antiviral and antimicrobial assessment of some selected flavonoids. *Zeitschrift für Naturforschung C* **61**, 632-638 (2006).

[38] Mitscher, L. A., Okwute, S. K., Gollapudi, S. R., Drake, S. & Avona, E. Antimicrobial pterocarpans of Nigerian *Erythrina mildbraedii*. *Phytochemistry* **27**, 3449-3452 (1988).

[39] Araya-Cloutier, C., Vincken, J.-P., van de Schans, M. G., Hageman, J., Schaftenaar, G., den Besten, H. M. & Gruppen, H. QSAR-based molecular signatures of prenylated (iso) flavonoids underlying antimicrobial potency against and membrane-disruption in Gram positive and Gram negative bacteria. *Scientific Reports* **8**, 9267 (2018).
